# Glycolytic interference blocks influenza A virus propagation by impairing viral polymerase-driven synthesis of genomic vRNA

**DOI:** 10.1371/journal.ppat.1010986

**Published:** 2023-07-13

**Authors:** Jens Kleinehr, Michael Schöfbänker, Katharina Daniel, Franziska Günl, Fakry Fahmy Mohamed, Josua Janowski, Linda Brunotte, Yvonne Boergeling, Marie Liebmann, Matthias Behrens, Andrea Gerdemann, Luisa Klotz, Melanie Esselen, Hans-Ulrich Humpf, Stephan Ludwig, Eike R. Hrincius

**Affiliations:** 1 Institute of Virology Muenster (IVM), Westfaelische Wilhelms-University Muenster, Muenster, Germany; 2 Department of Virology, Faculty of Veterinary Medicine, Zagazig University, Sharkia, Egypt; 3 Department of Neurology with Institute of Translational Neurology, University Hospital Muenster, Muenster, Germany; 4 Institute of Food Chemistry, Westfaelische Wilhelms-University Muenster, Muenster, Germany; Brigham and Women’s Hospital, UNITED STATES

## Abstract

Influenza A virus (IAV), like any other virus, provokes considerable modifications of its host cell’s metabolism. This includes a substantial increase in the uptake as well as the metabolization of glucose. Although it is known for quite some time that suppression of glucose metabolism restricts virus replication, the exact molecular impact on the viral life cycle remained enigmatic so far. Using 2-deoxy-d-glucose (2-DG) we examined how well inhibition of glycolysis is tolerated by host cells and which step of the IAV life cycle is affected. We observed that effects induced by 2-DG are reversible and that cells can cope with relatively high concentrations of the inhibitor by compensating the loss of glycolytic activity by upregulating other metabolic pathways. Moreover, mass spectrometry data provided information on various metabolic modifications induced by either the virus or agents interfering with glycolysis. In the presence of 2-DG viral titers were significantly reduced in a dose-dependent manner. The supplementation of direct or indirect glycolysis metabolites led to a partial or almost complete reversion of the inhibitory effect of 2-DG on viral growth and demonstrated that indeed the inhibition of glycolysis and not of *N*-linked glycosylation was responsible for the observed phenotype. Importantly, we could show via conventional and strand-specific qPCR that the treatment with 2-DG led to a prolonged phase of viral mRNA synthesis while the accumulation of genomic vRNA was strongly reduced. At the same time, minigenome assays showed no signs of a general reduction of replicative capacity of the viral polymerase. Therefore, our data suggest that the significant reduction in IAV replication by glycolytic interference occurs mainly due to an impairment of the dynamic regulation of the viral polymerase which conveys the transition of the enzyme’s function from transcription to replication.

## Author summary

Upon infection the influenza A virus alters the metabolism of infected cells. Among others, this includes a pronounced increase in glucose metabolism. We aimed to get a better understanding of these metabolic virus-host interactions and to unravel the mechanism by which glycolytic inhibition impairs the viral life cycle. On the one hand, we observed a virus-induced upregulation of many glycolysis metabolites which could often be reversed by the administration of a glycolysis inhibitor. On the other hand, our data suggested that the inhibitor treatment severely impaired viral propagation by interfering with the regulation of the viral polymerase. This manifested in an extended phase of transcription, while replication was strongly reduced. Additionally, we assessed the safety and tolerability of the used drug in immortalized and primary cells. Our study sheds more light on metabolic virus-host interactions and provides a better understanding of metabolic interference as a potential host-targeted antiviral approach, which does not bear the risk of creating resistances.

## 1. Introduction

Influenza viruses (IVs) still constitute a major risk factor for the human health all over the globe. According to extrapolations, 3–5 million severe cases and up to half a million deaths occur on average during annual IV epidemics [1]. The influenza A virus (IAV) is of special interest since it has zoonotic and pandemic potential. The high mutation rate of the IV genome easily allows to develop resistances to antiviral treatments. Therefore, more and more research focuses on targeting cellular factors, which are indispensable for viral replication, to develop novel host-targeted antiviral strategies. Since viruses in general are intracellular parasites and thus have no metabolism on their own, they completely depend on the host cell’s metabolism for their replication. Moreover, each type of virus reshapes the host cell’s metabolism towards its specific needs by regulating–often increasing–the uptake of metabolites and the activity of certain metabolic pathways [2–6]. Frequently, this includes elevated activity of glycolysis, the pentose phosphate pathway (PPP), lipid metabolism and the generation of amino acids [3]. This was also demonstrated for IV infections. Altered activity or elevated levels of pathway intermediates of, among others, glutaminolysis [7–9], fatty acid synthesis (FAS) [7,9], the PPP [7,8], the hexosamine biosynthetic pathway [9] and the tricarboxylic acid (TCA) cycle [7,8] were observed. However, especially an increased glycolytic rate and uptake of glucose has been described in various immortalized and primary cells after infection with IV as well as in the lungs of infected patients [7,8,10]. Direct inhibition of glycolysis or mediators of glycolysis led to a significant impairment of IV reproduction and spread [7,11,12]. Furthermore, the concentration of extracellular lactate increases during IV infections [8], suggesting the exploitation of aerobic glycolysis. This is indicative of the Warburg effect [13,14], in which cells metabolize glucose rather to lactate instead of pyruvate despite the adequate availability of oxygen. In this scenario, which is also observed in tumors, cells depend more on glycolysis than oxidative phosphorylation (OXPHOS) for sufficient synthesis of adenosine triphosphate (ATP). On the one hand IV benefits from inducing the Warburg effect by rapidly generating large amounts of biological building blocks for its replication and on the other hand by producing more lactate, which inhibits the induction of type I interferons [15], to counteract the immune response.

In our research we targeted the glucose metabolism with a special focus on the inhibition of glycolysis with the inhibitor 2-deoxy-d-glucose (2-DG), which has already been demonstrated to interfere with the formation of new infectious IV particles [11,12,16,17]. Beside the competitive inhibition of glucose uptake, 2-DG inhibits the first two glycolytic enzymes hexokinase (HK) and glucose-6-phosphate isomerase (GPI), the latter being its primary target. Just like glucose, 2-DG will be phosphorylated at the C6 position by HK to 2-deoxy-d-glucose-6-phosphate (2-DG-6-P). 2-DG-6-P competitively inhibits GPI and cannot be further metabolized by this enzyme. The increasing concentration of 2-DG-6-P leads to a feedback that additionally inhibits hexokinase in an allosteric manner [18–22]. Moreover, 2-DG gets fraudulently incorporated into oligosaccharide chains needed for *N*-linked glycosylation of glycoproteins [23], partially preventing this post-translational modification [24] and hence affecting the proteins’ folding and their functions. This inhibition is mainly conveyed by guanosine diphosphate (GDP)-2-DG into which 2-DG can be converted [25]. Thereby, 2-DG evidentially inhibits glycolysis and interferes with *N*-linked glycosylation. Here, we demonstrate the inhibitor’s significant impact on the replication of IAV without causing irreversible damage to the host cells. Furthermore, we unraveled a major mechanism by which this treatment interferes with the viral life cycle and discuss the potential of metabolic interference to fight severe IAV infections.

## 2. Results

### 2.1 2-DG is well tolerated in cells and exhibits strong virus-restricting activity

**Fig 1:**
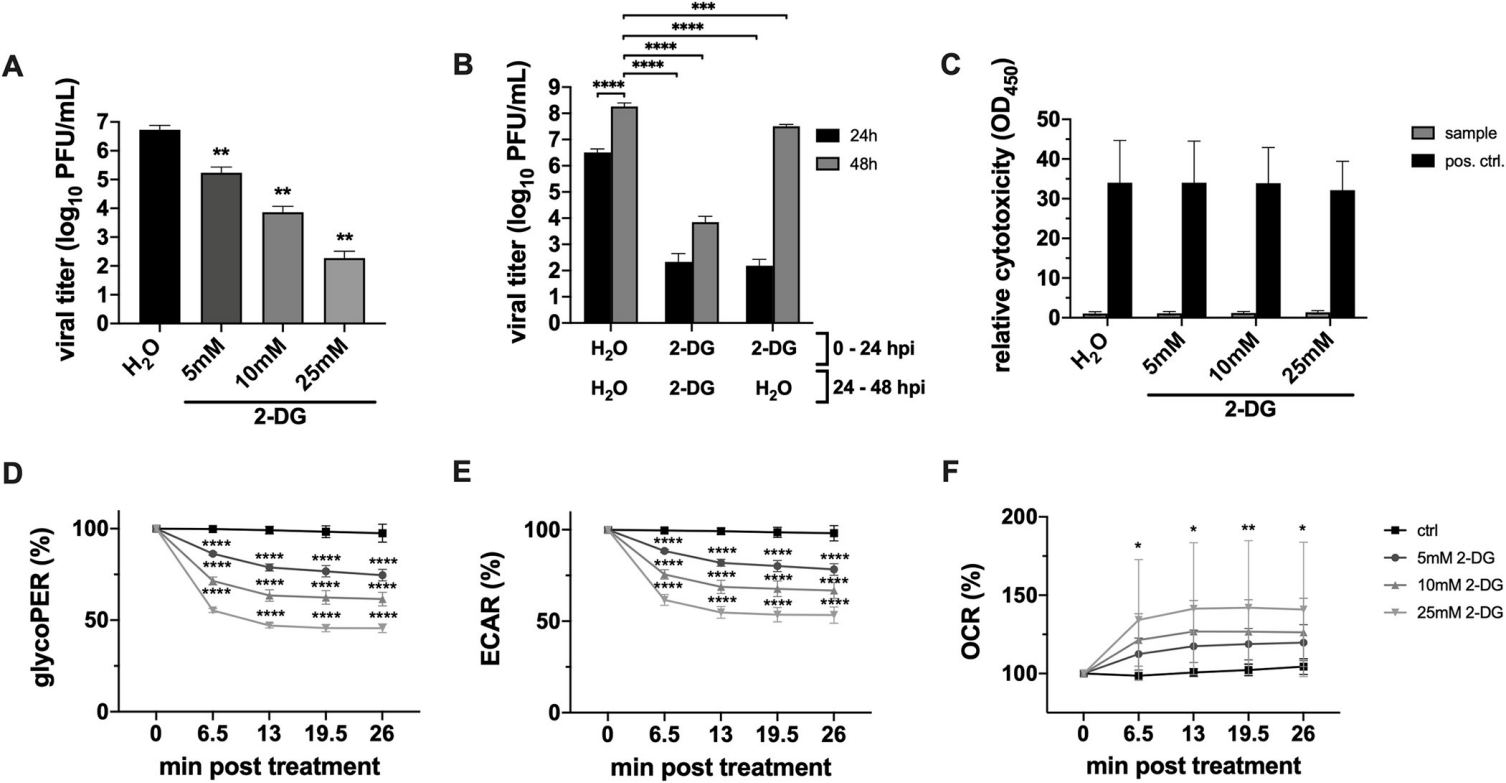
2-DG impairs IAV propagation and is well tolerated by A549 cells. **(A+B)** 24 h after seeding, A549 cells were infected with SC35M at an MOI of 0.001 for 30 min and were incubated in the presence of 25 mM glucose and the indicated concentrations of 2-DG or its solvent water for **(A)** 24 h or **(B)** 24 and 48 h. Subsequently, supernatants were collected to determine viral titers via plaque assay. **(C)** Uninfected cells were treated with the indicated inhibitor concentrations for 24 h and were then subjected to LDH assay for assessment of the relative cytotoxicity of the treatment. **(D-F)** The glycolytic proton efflux rate (glycoPER), extracellular acidification rate (ECAR) and oxygen consumption rate (OCR) were measured in real-time via glycolytic rate assay in a Seahorse XFe96 Analyzer. The kinetics show the influence of different concentrations of 2-DG on the three measured parameters. Depicted are the means *±* SD of **(A-C)** three or **(D-F)** five independent experiments with **(A-C)** three or **(D-F)** four biological replicates per condition and experiment. Statistical significances were determined via **(A)** unpaired one-way ANOVA and Dunnett’s correction, comparing all treated samples to the water control and **(B-F)** ordinary two-way ANOVA with **(B)** Tukey’s, **(C)** Sidak’s and **(D-F)** Dunnett’s correction for multiple comparison, comparing **(B)** all samples with one another, **(C)** all treated samples of one group to the respective water control or **(D-F)** the time points of differentially treated cells with their respective start value. p-values are indicated as follows: < 0.05 = *, < 0.01 = **, < 0.001 = ***, < 0.0001 = ****.

Our first aim was to prove the virus-restricting potential of 2-DG in cell culture. First, we showed in plaque assays that the number of newly produced infectious IAV particles decreased significantly in a dose-dependent manner when 2-DG was applied directly after the infection of A549 cells with the recombinant H7N7 strain A/Seal/Massachusetts/1/80 (SC35M) (**Fig 1A**). This decrease became as strong as more than four orders of magnitude when the glucose/2-DG ratio was 1:1. Second, we observed a very similar 2-DG-mediated decrease for IAV nucleoprotein (NP)-positive cells via flow cytometry (**S1A Fig**). These data demonstrated the strong impairment of IAV reproduction and spread in the presence of 2-DG. Next, we assessed the reversibility as well as metabolic and potential cytotoxic effects of the 2-DG treatment on cells. Here, it could be demonstrated that the strong antiviral effect of a 24 h treatment was quickly abolished once the inhibitor was removed (**Fig 1B**). The massive increase of viral titers after the replacement of 2-DG with inhibitor-free medium suggested the full reversibility of 2-DG-induced effects and indicated that there was no permanent cell damage which is also substantiated by the literature [11]. By performing lactate dehydrogenase (LDH) assays we detected no cytotoxicity within the range of used 2-DG concentrations (**Fig 1C**), as previously demonstrated in various cell lines including A549 [26,27]. Moreover, we could even observe a beneficial effect of the 2-DG treatment for the survival of infected cells. With increasing 2-DG concentrations the total percentage of dead cells decreased significantly 24 hours post infection (hpi) (**S1B Fig**). However, the results of the LDH assays in combination with data obtained from trypan blue exclusions suggested a certain cytostatic effect, since even though the viability of all samples was not affected, total cell counts decreased with rising 2-DG concentrations (**S1C and S1D Fig**). In line with these results, a cytostatic effect of 2-DG has also been observed previously in other cells [27–29]. Furthermore, we investigated the effect of 2-DG on the metabolism in real-time via a Seahorse Analyzer. We observed a very rapid and significant reduction of the glycolytic proton efflux rate (glycoPER), which constitutes a direct read-out of the glycolytic rate (**Fig 1D**), as well as the extracellular acidification rate (ECAR) (**Fig 1E**). Simultaneously, the oxygen consumption rate (OCR) of 2-DG-treated cells increased quickly after the treatment (**Fig 1F**). These data proved the partial inhibition of glycolysis by 2-DG and indicated that cells were able to compensate the loss of glycolytic activity by upregulating cellular respiration to generate energy. Since 2-DG also influenced the OCR and consequentially OXPHOS, we tested the effect of oligomycin A, an OXPHOS inhibitor, on IAV replication and on the treated cells (**S2 Fig**). The oligomycin A treatment had a strong antiviral effect which was even increased in combination with 2-DG (**S2A Fig**). The combination of the two substances also increased the cytostatic effect, shown by significantly reduced cell counts (**S2B Fig**) but unaffected viability of treated cells (**S2C Fig**).

In addition to evaluating the cytotoxicity of 2-DG, we also tested potential effects of 2-DG on the innate immune response and the cellular responsiveness to viral infections. For that purpose, we measured expression levels of the proinflammatory genes *interleukin-6* (*IL-6*) and *C-X-C motif chemokine ligand 8* (*CXCL8*, protein: IL-8) as well as the interferon-stimulated genes (ISGs) *DExD/H-box helicase 58* (*DDX58*, protein: retinoic acid inducible gene I) and *myxovirus resistance gene A* (*MxA*) after stimulation with either cellular or viral RNA in the presence or absence of 2-DG (**S3A–S3D Fig**). We observed a mild to more pronounced induction of *IL-6* (**S3A Fig**) and *CXCL8* (**S3B Fig**) with increasing concentrations of 2-DG. This finding was consistent with a previous publication, reporting that nutrient shortage (also induced by 2-DG) triggers a cell response which resembles wound healing processes in cancer cells as well as in primary cells [26]. Moreover, the mild induction of proinflammatory cytokines in the presence of 2-DG might be attributed to the fact that the inhibitor can also impair glycosylation. This in turn gives rise to endoplasmic reticulum (ER) stress, elicited by deficient glycoproteins, consequently leading to the unfolded protein response (UPR) [23] which has been demonstrated to drive the production of proinflammatory cytokines [30]. On the other hand, we measured no clear differences in the expression of *DDX58* (**S3C Fig**) and *MxA* (**S3D Fig**) in the presence of lower 2-DG concentrations but a moderate and significant reduction of both ISGs at 25 mM of the inhibitor, when stimulated with viral RNA. Nevertheless, our data confirmed that the cells were well responsive to viral stimuli, regardless of the concentration of 2-DG that was applied.

Apart from the permanent cell line A549, key experiments were repeated in primary human bronchial epithelial cells (HBEpCs) and genuine human lung explants (**S4A–S4E Fig**). Since the used media for primary cells and primary tissue contained less glucose, lower concentrations of the inhibitor were used. However, we still applied the same 2-DG/glucose ratio to human lung explants as in A549 experiments which led to a significant and dose-dependent reduction of viral titers (**S4A Fig**). Because HBEpCs were more susceptible to the treatment, lower 2-DG/glucose ratios were applied. The highest concentration used in HBEpC experiments was 1200 μM which corresponds to the 2-DG/glucose ratio (1:5) of 5 mM 2-DG in experiments carried out with A549 cells. Similar to A549 cells, HBEpCs displayed barely any signs of cytotoxicity after treatment (**S4B Fig**). Reduced lactate concentrations in the supernatant of treated cells indirectly indicated the efficiency of glycolytic inhibition (**S4C Fig**). Importantly, the treatment with 2-DG also led to a significant and dose-dependent reduction of viral titers in HBEpCs (**S4D and S4E Fig**). Even though the magnitude of the inhibitory effect on glycolysis and viral replication differed slightly from the data obtained with A549 cells–most likely due to distinct cellular metabolic activities and lower 2-DG/glucose ratios (HBEpC)–these data suggested the safe use and antiviral activity of 2-DG in primary tissue.

### 2.2 2-DG moderately affects viral protein translation in a single viral life cycle

**Fig 2:**
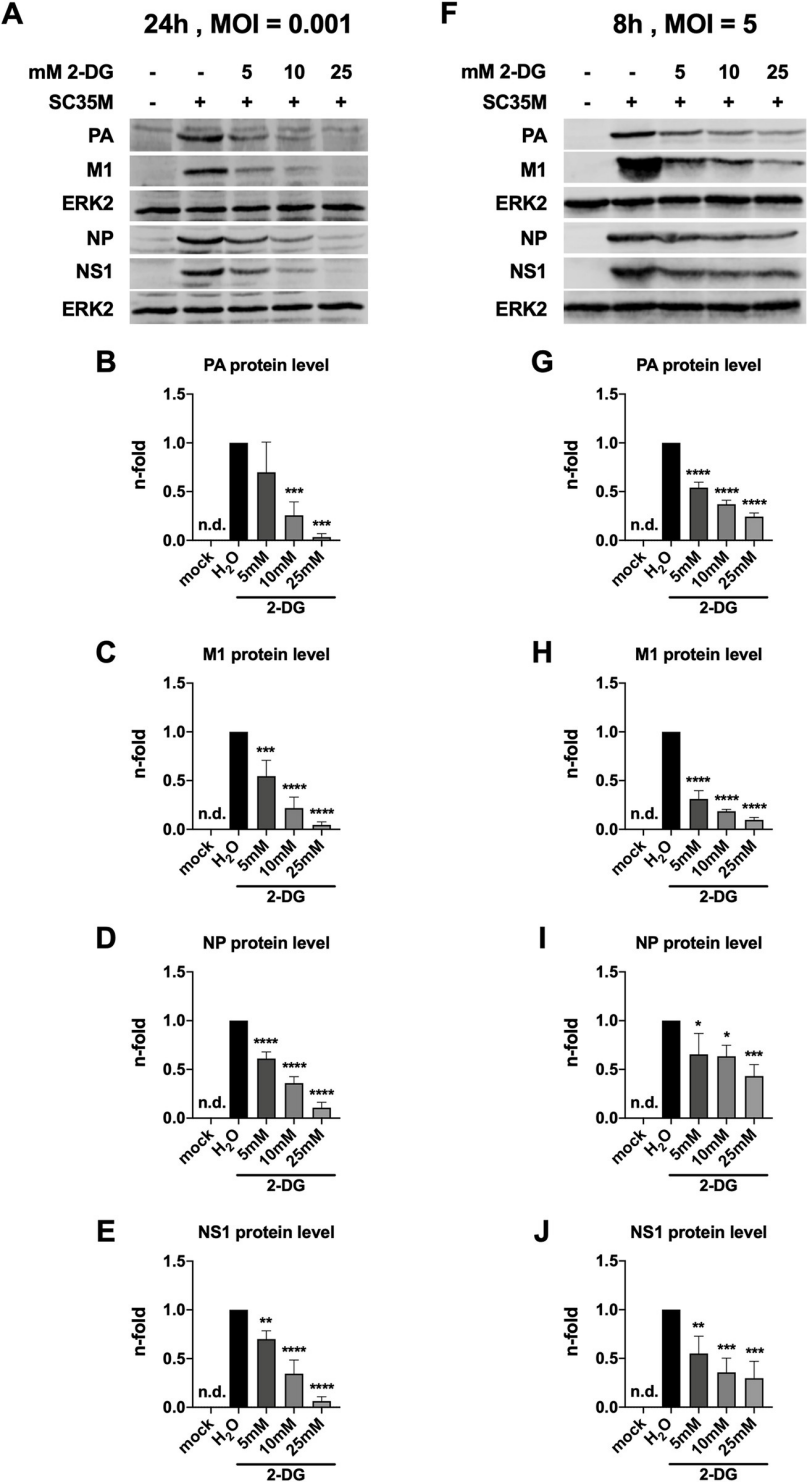
2-DG-mediated reduction of viral protein expression. 24 h after seeding, A549 cells were infected with SC35M at the depicted MOIs for 30 min and were incubated with 25 mM glucose and the indicated concentrations of 2-DG for a total of **(A-E)** 24 h or **(F-J)** 8 h. Protein lysates of triplicates were unified to yield sufficient protein amounts. Proteins were separated via SDS-PAGE. Visualization was done using primary antibodies against PA (rabbit), M1 (mouse), NP (rabbit), NS1 (rabbit) and ERK2 (rabbit) and fluorescence-labelled anti-mouse (donkey) and anti-rabbit (donkey) secondary antibodies. Depicted are representative protein bands from one out of three independent experiments. **(B-E, G-J)** Densitometric analyses were performed to quantify protein accumulation by first normalizing viral proteins to the loading control ERK2 and then normalizing all other samples to the infected but untreated sample. Depicted are the means *±* SD of three independent experiments. Statistical significances were determined via unpaired one-way ANOVA and Dunnett’s correction, comparing all other samples to the infected but untreated sample (second lane). p-values are indicated as follows: < 0.05 = *, < 0.01 = **, < 0.001 = ***, < 0.0001 = ****.

Given the remarkable impairment of IAV replication by 2-DG, we now aimed to identify the spot of interference of the drug within the viral life cycle. Therefore, we checked potential changes in the accumulation of various IAV proteins 24 hpi (**Fig 2A–2E**) and after a single replication cycle of 8 h (**Fig 2F–2J**). In accordance with the strongly reduced viral titers there was also a severe reduction of viral protein accumulation after 24 h. Within a single replication cycle we detected less pronounced but still significant differences in viral protein accumulation between differently treated samples. The accumulation of polymerase acidic protein (PA) and matrix protein 1 (M1) was stronger impaired than the accumulation of NP and non-structural protein 1 (NS1). The reduction of viral proteins within a single replication cycle suggested reduced viral protein accumulation to be partially the reason for the severe impact of 2-DG on IAV propagation.

To rule out a general effect on the cellular translation machinery, we measured the fluorescence signal of the reporter *Renilla* luciferase, driven by a constitutive promoter, in a luciferase assay in the absence or presence of various concentrations of 2-DG (**S5A Fig**). Decreased signals would be an indication for an impairment of cellular transcription and/or translation. Interestingly, there was no negative effect on the luciferase signal, suggesting no general impairment of the cellular protein synthesis. Quite the opposite was the case when high concentrations of 2-DG were used which even led to an increase of the luciferase signal. To further verify these results several typical cellular proteins were detected via western blot after the treatment with different concentrations of 2-DG (**S5B–S5F Fig)**. While the viral protein M1 was heavily decreased in the presence of 2-DG, none of the cellular proteins was significantly affected. Additionally, we examined the possible involvement of an altered turnover of viral mRNA or proteins mediated by 2-DG. For that we compared the mRNA levels of M1 and the protein accumulation of PA and M1 in the absence and presence of either the transcription inhibitor actinomycin D or the proteasome inhibitor MG132 (**S5 Fig**). In the presence of actinomycin D, the turnover of M1 mRNA was comparable between untreated and 2-DG-treated samples (**S6A Fig**). Whether MG132 was applied or not, very similar trends of a 2-DG-mediated reduction of viral protein accumulation were seen (**S6B–S6D Fig**). This indicated that the turnover of viral mRNA and proteins was not affected by 2-DG.

### 2.3 Glycolytic interference prolongs the phase of viral transcription while it clearly reduces viral replication within a replication cycle

After analyzing the effect of 2-DG on viral protein accumulation, we delved deeper into the IAV replication cycle to understand the virus-restricting properties of 2-DG. Therefore, we now examined if a treatment with 2-DG interfered with the main processes driven by the viral polymerase: transcription and replication. Since IAV is a negative-sense RNA virus its RNA-dependent RNA polymerase can, right after reaching the host cell’s nucleus, transcribe positive-sense mRNA. After translation and nuclear import, nascent viral polymerase complexes mediate the two-step process of replication. Here, a positive-sense, full-length complementary RNA (cRNA) is synthesized from the initial viral genomic RNA (vRNA) which subsequently serves as a template for vRNA synthesis [31,32].

**Fig 3:**
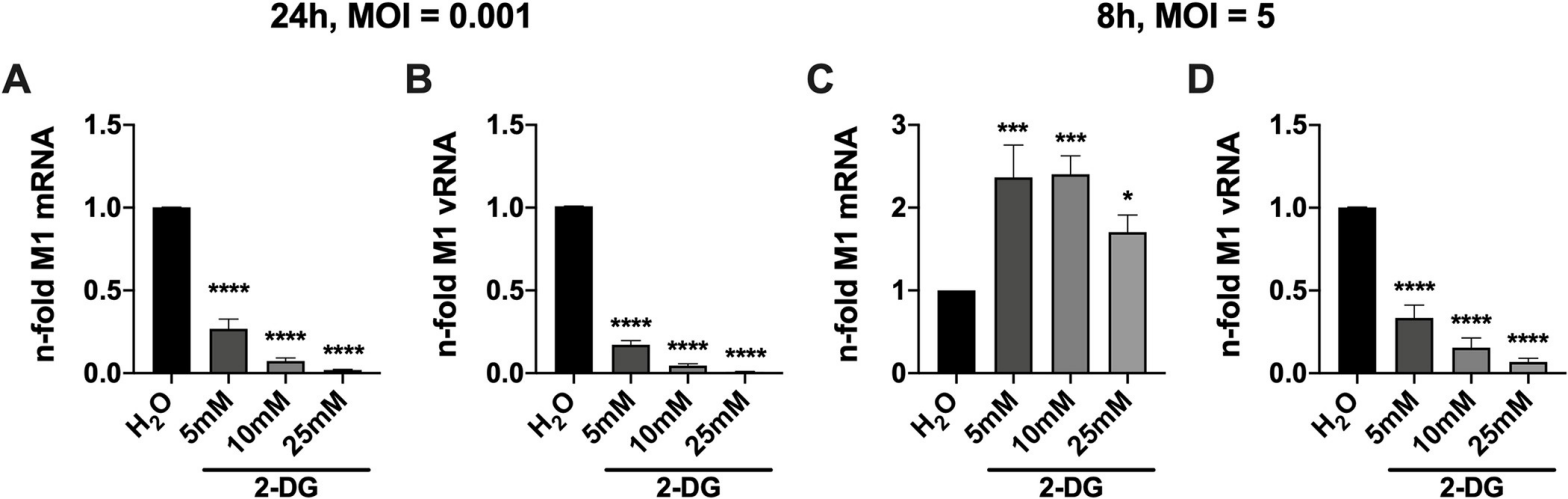
2-DG conversely affects IAV mRNA and vRNA accumulation. 24 h after seeding, A549 cells were infected with SC35M at the depicted MOIs for 30 min and were incubated with 25 mM glucose and the indicated concentrations of 2-DG for a total of **(A+B)** 24 h or **(C+D)** 8 h. Subsequently, cells were lysed, their RNA isolated and cDNA synthesized using either **(A+C)** oligo(dT) primers to transcribe mRNA or **(B+D)** fluA uni12 primers to transcribe vRNA. Real-time qPCR was performed with two technical replicates per sample and values of treated samples were normalized to the water control. In case of mRNA detection, all results were additionally normalized to a GAPDH control. Depicted are the means ± SD of three independent experiments with three biological replicates per condition and experiment. Statistical significances were determined via unpaired one-way ANOVA and Dunnett’s correction, comparing all treated samples to the water control. p-values are indicated as follows: < 0.05 = *, < 0.01 = **, < 0.001 = ***, < 0.0001 = ****.

We analyzed the accumulation of viral mRNA and vRNA that codes for M1. In case of vRNA detection, the values of M1 are representative of segment 7 (M). As before, M1 mRNA and vRNA were analyzed after 24 h (**Fig 3A and 3B**) and after a single replication cycle of 8 h (**Fig 3C and 3D**) with and without 2-DG. As observed for viral proteins, we measured a massive reduction of M1 mRNA and vRNA 24 hpi when 2-DG was applied (**Fig 3A and 3B**), which is in line with the reduction of viral titers. Experiments for the duration of a single replication cycle, however, revealed intriguing differences between the two distinct RNA species. While viral mRNA levels were elevated in the presence of 2-DG (**Fig 3C**) the amount of vRNA was clearly reduced after an infection period of 8 h (**Fig 3D**). Again, these experiments were repeated with HBEpCs to see if there are similar effects in non-transformed cells with no altered metabolism (**S4F–S4I Fig**). Using these primary cells, we observed a very similar pattern of IAV mRNA and vRNA accumulation through the treatment with 2-DG as in A549 cells. While mRNA was decreased 24 hpi (**S4F Fig**) and unaffected 8 hpi (**S4G Fig**), vRNA was decreased at both time points (**S4H and S4I Fig**). To further examine the similarities and difference between A549 cells and primary cells, another cell line, Calu-3, was used in a similar experiment. 8 hpi we observed moderately decreased levels of viral mRNA accumulation (**S7A and S7B Fig**) and again severely decreased levels of vRNA (**S7C and S7D Fig**). The slight differences in mRNA accumulation between the here used cell systems occurred likely due to cell-specific effects, e.g., different virus replication dynamics, or a milder 2-DG treatment in the case of HBEpCs. Nevertheless and most importantly, the strong reduction in vRNA accumulation, limiting viral propagation, seemed to be tissue-independent.

With this phenotype at hand, we wanted to exclude a virus strain-specific effect and additionally analyzed the influence of 2-DG on viral growth, transcription, and replication of the recombinant H3N2 strain A/Panama/2007/1999 (Pan/99). As for SC35M, we observed a strong dose-dependent decrease of viral titers, mRNA and vRNA 24 hpi (**S8A–S8C Fig).** Importantly, with an increase of Pan/99 mRNA and a decrease of vRNA in a single cycle experiment **(S8D and S8E Fig)** the results resembled those obtained with SC35M. Therefore, glycolytic interference on IAV appears to be a general phenomenon and not a virus strain-specific effect.

Summing up the obtained insights, the qPCR data suggested that the main cause for the impairment of IAV reproduction and spread by 2-DG is the interference of the inhibitor with the production of viral genome copies. Hereafter, we were especially interested in why glycolytic inhibition barely affected or even increased viral mRNA but always decreased vRNA within a single viral life cycle in various cell lines.

**Fig 4:**
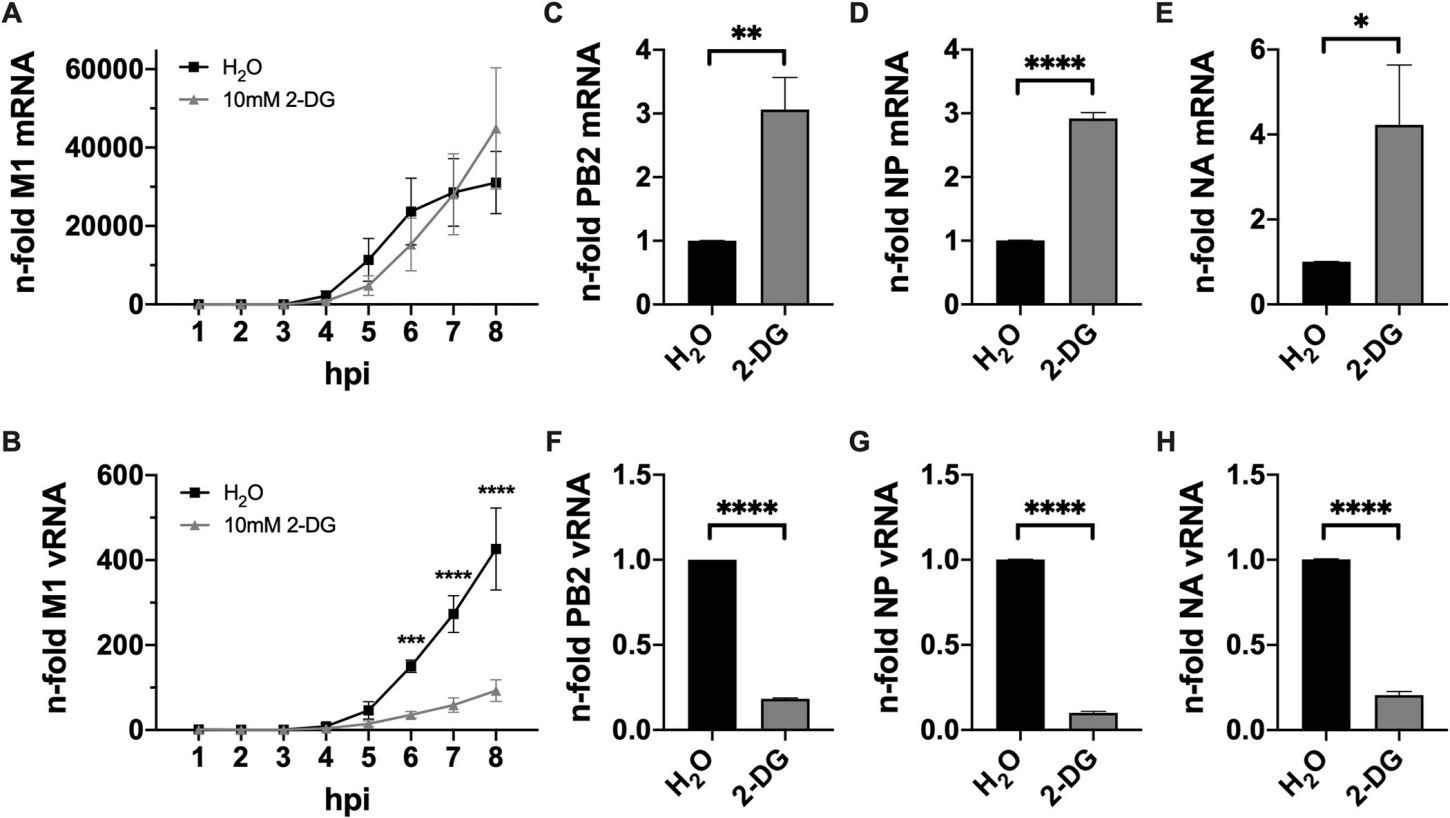
Prolongation of IAV transcription and reduction of replication by 2-DG. 24 h after seeding, A549 cells were infected with SC35M at an MOI of 5 for 30 min and were incubated without or with 10 mM 2-DG in the presence of 25 mM glucose for a total of 8 h. **(A+B)** Each hour or **(C-H)** 8 hpi cells were lysed, their RNA isolated and cDNA synthesized using **(A)** oligo(dT) primers, **(B)** fluA uni12 primers or **(C-H)** specific primers to transcribe mRNA and vRNA of the SC35M gene segments 1 (PB2), 5 (NP) and 6 (NA). Real-time qPCR was performed with two technical replicates per sample. **(A+B)** All values were normalized to the water control 1 hpi or **(C-H)** values of treated samples were normalized to the water control. Depicted are the means *±* SD of three independent experiments with three biological replicates per condition and experiment. Statistical significances were determined **(A+B)** via ordinary two-way ANOVA and Sidak’s correction, comparing the treated sample of each time point to its respective water control or **(C-H)** via unpaired t-test. p-values are indicated as follows: < 0.05 = *, < 0.01 = **, < 0.001 = ***, < 0.0001 = ****.

In order to shed light on this question we performed an 8 h infection kinetic and analyzed the synthesis of M1 mRNA and vRNA in the presence of 2-DG in comparison to an untreated control (**Fig 4A and 4B**). In untreated cells the production of viral mRNA reached its strongest incline at approximately 6 hpi and started to establish a plateau afterwards (**Fig 4A**, black line). In contrast, the treatment with 2-DG led to a slower but continuous increase of mRNA transcription, eventually exceeding the total accumulation of viral mRNA in untreated cells (**Fig 4A**, gray line). Thus, despite a lower accumulation rate of viral mRNA in treated cells in the first 6 h of an infection, these samples displayed higher mRNA levels at time points later than 7 hpi. Even though the underlying mechanisms are unknown this observation explained why we detected higher viral mRNA levels in 2-DG-treated cells after one replication cycle (**Fig 3C**). In accordance with our previous data on vRNA accumulation at 8 hpi (**Fig 3D**), the kinetic revealed that vRNA accumulated at a clearly reduced rate when 2-DG was applied throughout the whole experiment (**Fig 4B**, gray line). Besides, the exact same raw data were normalized to the water control of each individual time point to visualize the time-dependent differences between untreated and 2-DG-treated samples–especially the slower but prolonged accumulation rate of viral mRNA–more clearly (**S9 Fig**). To verify the results of **Fig 4A and 4B**, we performed strand-specific real-time qPCR according to the protocol established by Kawakami *et al*. [33] for segment 5 (NP) and 6 (NA) with specific primers (**Table 1**). Additionally, we analyzed segment 1 (PB2), which is the longest of the IAV gene segments, to rule out effects which might be caused by the length of different segments. We determined the n-fold of viral mRNA and vRNA of the three segments in 2-DG-treated cells 8 hpi in comparison to untreated cells. The results for all three gene segments were very similar and supported the previous kinetics. We observed a 3-4-fold increase of viral mRNA (**Fig 4C–4E**) while the vRNA of the same gene segments was decreased by approximately 80–90% (**Fig 4F–4H**) when 2-DG was applied. Notably, these findings confirmed our previous measurements of mRNA and vRNA after one replication cycle (**Fig 3C and 3D**). The data presented in **Fig 4** indicated that glycolytic inhibition by 2-DG prolonged the phase of viral mRNA transcription while it attenuated viral genome replication. This suggested either a distinct effect on the transcriptional and replicative capacity of the viral polymerase or an impairment of the dynamic regulation of the polymerase function, determining whether it performs transcription or replication.

**Table 1:** Primer for strand-specific real-time qPCR subdivided into their use in reverse transcription and PCR.

target		purpose	primer name	sequence (5’ - 3’)	position (nt)
SC35M segment 1 (PB2)	vRNA	RT	vRNAtag_SC35M_seg1_1540F	GGCCGTCATGGTGGCGAAT CGGGATCAACGAGGGAATGTACTAC	1540–1564
		PCR	vRNAtag	GGCCGTCATGGTGGCGAAT	
			SC35M_seg1_1704R	AGTTTCCCAGTTCCTGATGATCCA	1704–1681
	cRNA	RT	cRNAtag_SC35M_seg1_2341R	GCTAGCTTCAGCTAGGCATC AGTAGAAACAAGGTCGTTTTTAAAC	2341–2317
		PCR	cRNAtag	GCTAGCTTCAGCTAGGCATC	
			SC35M_seg1_2176F	GCGAAGGGAGAGAAGGCTAATGTGC	2176–2200
	mRNA	RT	mRNAtag_SC35M_seg1_dTR	CCAGATCGTTCGAGTCGT TTTTTTTTTTTTTTTTAAACAATTCGA	2325–2310
		PCR	mRNAtag	CCAGATCGTTCGAGTCGT	
			SC35M_seg1_2176F	GCGAAGGGAGAGAAGGCTAATGTGC	2176–2200
SC35M segment 5 (NP)	vRNA	RT	vRNAtag_SC35M_seg5_675F	GGCCGTCATGGTGGCGAAT AAATGGGCGGAGAACAAGAATTGC	675–698
		PCR	vRNAtag	GGCCGTCATGGTGGCGAAT	
			SC35M_seg5_845R	CTCAGAATGAGAGCAGACCGTGCA	845–822
	cRNA	RT	cRNAtag_SC35M_seg5_1565R	GCTAGCTTCAGCTAGGCATC AGTAGAAACAAGGGTATTTTTCTTT	1565–1541
		PCR	cRNAtag	GCTAGCTTCAGCTAGGCATC	
			SC35M_seg5_1466F	CGATCGTGCCTTCCTTTGACATG	1466–1488
	mRNA	RT	mRNAtag_SC35M_seg5_dTR	CCAGATCGTTCGAGTCGT TTTTTTTTTTTTTTTTCTTTAATTGTT	1549–1534
		PCR	mRNAtag	CCAGATCGTTCGAGTCGT	
			SC35M_seg5_1466F	CGATCGTGCCTTCCTTTGACATG	1466–1488
SC35M segment 6 (NA)	vRNA	RT	vRNAtag_SC35M_seg6_734F	GGCCGTCATGGTGGCGAAT GTAGTGATGACCGATGGATCAGCA	734–757
		PCR	vRNAtag	GGCCGTCATGGTGGCGAAT	
			SC35M_seg6_885R	CAAGTTACTTTTGAATCGTGCCCATAG	885–859
	cRNA	RT	cRNAtag_SC35M_seg6_1413R	GCTAGCTTCAGCTAGGCATC AGTAGAAACAAGGGTGTTTTTGCAA	1461–1437
		PCR	cRNAtag	GCTAGCTTCAGCTAGGCATC	
			SC35M_seg6_1338F	GGTGGACGAGCAACAGCTTAGTTGC	1338–1362
	mRNA	RT	mRNAtag_SC35M_seg6_dTR	CCAGATCGTTCGAGTCGT TTTTTTTTTTTTTTTTGCAATTTACGA	1445–1430
		PCR	mRNAtag	CCAGATCGTTCGAGTCGT	
			SC35M_seg6_1338F	GGTGGACGAGCAACAGCTTAGTTGC	1338–1362
WSN segment 6 (NA)	vRNA	RT	vRNAtag_WSN_seg6_689F	GGCCGTCATGGTGGCGAAT ACCATAATGACCGATGGCCCAAGT	689–712
		PCR	vRNAtag	GGCCGTCATGGTGGCGAAT	
			WSN_seg6_839R	ACATCACTTTGCCGGTATCAGGGT	839–816
	cRNA	RT	cRNAtag_WSN_seg6_1413R	GCTAGCTTCAGCTAGGCATC AGTAGAAACAAGGAGTTTTTTGAAC	1413–1389
		PCR	cRNAtag	GCTAGCTTCAGCTAGGCATC	
			WSN_seg6_1314F	TGAATAGTGATACTGTAGATTGGTCT	1314–1339
firefly (FF)	vRNA	RT	tag-vRNA-FF	GGCCGTCATGGTGGCGAAT GGGTCACCTAAGGGTGTGGCCC	
		PCR	vRNAtag	GGCCGTCATGGTGGCGAAT	
			vRNA-FF-rev	CCAAAACCGTGATGGAATGGAACAACA	
	mRNA	RT	tag-mRNA-FF	CCAGATCGTTCGAGTCGT TTTTTTTTTTTTTCTTACACGGCGATC	
		PCR	mRNAtag	CCAGATCGTTCGAGTCGT	
			mRNA/cRNA-FF-fwd	GGATTACGTCGCCAGTCAAG	

### 2.4 2-DG treatment does not affect the replicative capacity of the viral polymerase nor the durability of RNP complexes

**Fig 5:**
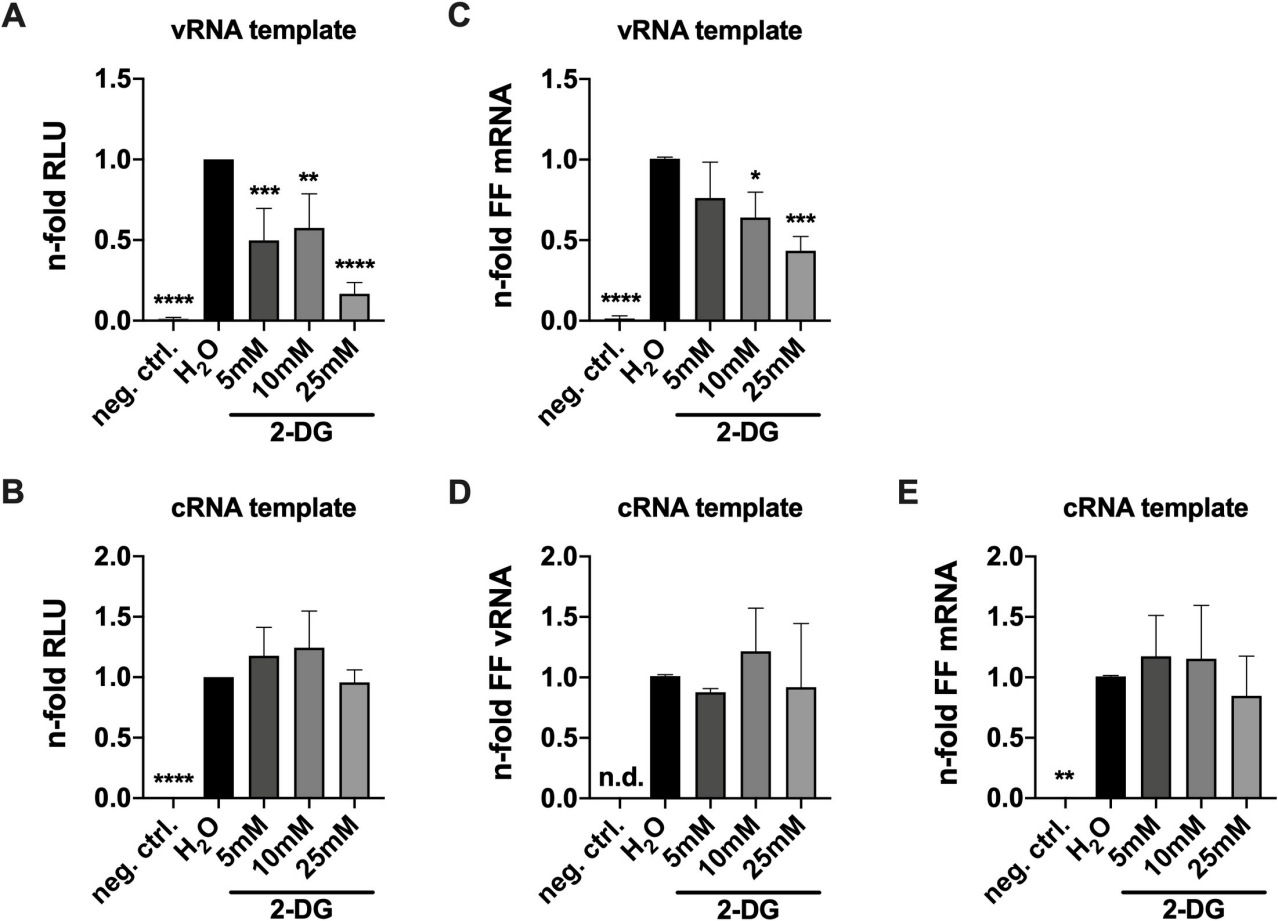
2-DG shows no effect on the replicative capacity of the IAV polymerase. 24 h after seeding, HEK293T cells were transfected with plasmids encoding PA, PB1, PB2 and NP of SC35M and the transfection control Renilla luciferase and either a **(A+C)** vRNA-like or **(B,D+E)** cRNA-like template of the Firefly (FF) luciferase. The negative control was transfected with an empty vector instead of PB2. 4 h later the transfection solution was replaced with medium containing 25 mM glucose and the indicated concentrations of 2-DG for another 20 h. Subsequently, cells were lysed and **(A+B)** relative light units (RLU) were measured via luciferase assay or **(C-E)** RNA accumulation was analyzed via qPCR. **(A+B)** All values were normalized to their respective transfection control. **(A-E)** The n-folds were calculated in regard to each water control. Statistical significances were determined via unpaired one-way ANOVA and Dunnett’s correction, comparing all other samples to the water control. p-values are indicated as follows: < 0.05 = *, < 0.01 = **, < 0.001 = ***, < 0.0001 = ****.

After revealing that reduced vRNA accumulation in the presence of 2-DG was the most crucial consequence of glycolytic interference for viral growth, we wanted to understand this phenomenon more mechanistically. Minigenome systems can be used to explicitly focus on transcription and replication without the dynamic of a full-fledged infection and hence allow to dissect distinct steps of the viral life cycle to a certain degree. Here, minigenome assays were performed as described previously [34] to assess whether 2-DG has a direct influence on the activity of the viral polymerase. For this purpose, we transfected HEK293T cells with plasmids encoding all proteins of the viral ribonucleoprotein (vRNP) complex–PA, PB1, PB2 and NP–together with a reporter plasmid coding for a *Firefly* (FF) luciferase under the control of a viral promoter. Another plasmid that constitutively expressed *Renilla* luciferase was co-transfected to serve as a transfection control. Subsequently, those cells were mock-treated or treated with 2-DG and analyzed via luciferase assay. By transfecting two different expression plasmids of the FF reporter luciferase either vRNA-like or cRNA-like RNA templates were synthesized, which were converted by the transfected and nascent viral proteins. Thus, we were able to analyze the effect of 2-DG on the transcriptional capacity of the viral polymerase (**Fig 5A**) or a potential effect on the replicational capacity of the polymerase since vRNA first had to be synthesized from the cRNA-like template (**Fig 5B**). We observed that transcription was significantly reduced in the presence of 2-DG (**Fig 5A**) which confirms the previously seen 2-DG-induced lower accumulation rate of viral mRNA in the earlier phase of the 8 h kinetic (**Figs 4A and S9A**). On the other hand, there was no significant difference of the luciferase signal between the various samples when the cRNA plasmid was transfected (**Fig 5B**). These data were additionally verified via qPCR analyses of the luciferase reporter FF. This gave us a more direct readout and made it possible to discriminate between vRNA and mRNA synthesis after the transfection of the cRNA plasmid. The PCR data confirmed the luciferase data by also showing a reduction of transcription when the vRNA plasmid was transfected (**Fig 5C**) and no effect on replication when the cRNA plasmid was transfected (**Fig 5D**) through a treatment with 2-DG. Interestingly, mRNA synthesis was unaffected by 2-DG after the transfection of the cRNA plasmid (**Fig 5E**), which remained an unsolved observation so far. These data suggested no direct reduction of the replicational capacity of the viral polymerase by 2-DG. This could be confirmed in another experiment, in which a replication-competent but transcription-deficient PB2 (PB2-361A) [35,36], which we named PB2 R+/T-, was transfected. The replication competence and transcription deficiency were demonstrated by comparable vRNA values of FF between the wild type PB2 and PB2 R+/T- (**S10A Fig**) and FF mRNA n-folds comparable to the negative control when PB2 R+/T- was transfected (**S10B Fig**). PCR again revealed no difference of the replicational capacity of the viral polymerase through a 2-DG treatment (**S10A Fig**).

Since data from minigenome assays are rather suggestive compared to analyses of real infections we performed further strand-specific qPCRs in which we checked the accumulation of mRNA, cRNA and vRNA of various IAV segments (**S11 Fig**). Similar to the data from **Fig 4**, a strong impairment of vRNA accumulation could be observed (**S11G–S11I Fig**). Additionally, the previous step of replication, cRNA synthesis, seemed to be inhibited as well (**S11D–S11F Fig**), highlighting that initial synthesis of cRNA from vRNA was already affected by the 2-DG treatment. The accumulation of mRNA (**S11A–S11C Fig**) also followed the previously shown pattern. mRNA accumulated at a lower rate in the presence of 2-DG but constantly continued to increase and eventually surpassed the values of untreated cells. These data confirmed our previous results and showed that replication is already affected at the step of cRNA synthesis.

Additionally, we examined whether the 2-DG treatment potentially affected the durability (e.g., altered stability or rate of degradation) of RNP complexes and performed an assay based on a previous publication [37] in which HEK293T cells were pre-transfected with plasmids encoding all RNP complex proteins of SC35M. 24 h later they were infected with IAV and subsequently treated with 2-DG and cycloheximide, an inhibitor of translation, for 6 h. This way, the pre-transfected RNP proteins were synthesized and, after IAV infection, formed RNP complexes with the nascent cRNA and vRNA. Strand-specific real-time qPCR revealed that levels of cRNA and vRNA remained equal between the solvent control and 2-DG-treated samples (**S12A and S12B Fig**), which indicated no effect of 2-DG on the durability of RNP complexes. The experiment was repeated with the same plasmids of the H1N1 strain A/WSN/1933 (WSN), including a catalytically inactive PB1 (PB1- D445A/D446A) [37], which we named PB1(-), to eliminate potential effects of non-specific mRNA production (**S12C and S12D Fig**). These results confirmed the previous ones and showed no significant difference between the solvent control and 2-DG-treated samples. The fact that the vRNA values of our target samples did not surpass the ΔWSN-PA control, proved that polymerases with PB1(-) were unable to synthesize RNA (**S12D Fig**).

The data presented so far suggested that 2-DG mainly impaired IAV replication and spread by interfering with viral genome replication which was marked by massively reduced levels of cRNA and vRNA if the inhibitor was applied. However, minigenome assays suggested that 2-DG neither had a direct effect on the replicative capacity of the viral polymerase (**Fig 5C and 5D**) nor on the durability of vRNP complexes (**S12 Fig**). The data indicated a 2-DG-mediated disruption of the polymerase regulation since transcription was particularly extended while replication was reduced.

### 2.5 IAV infections and glycolytic interference alter the metabolic profile of A549 cells

**Fig 6:**
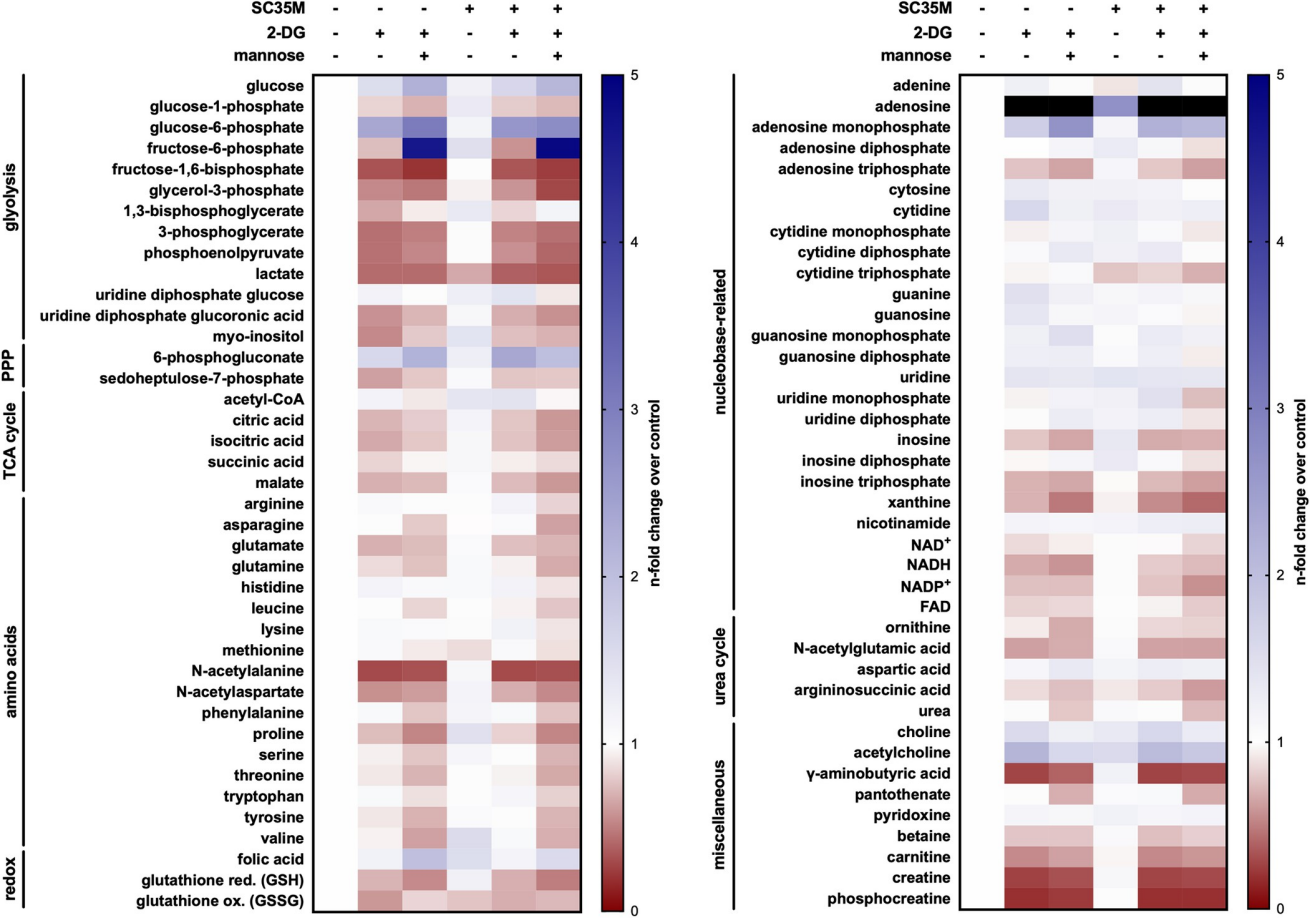
Metabolic alterations induced by IAV infection and glycolytic treatment within 8 h. A549 cells were mock-infected or infected with SC35M at an MOI of 5 and were subsequently incubated in DMEM (containing 25 mM glucose) with or without 10 mM 2-DG and 1 mM mannose as indicated. 8 hpi metabolic activity was quenched and intracellular metabolites were relatively quantified via HILIC-MS/MS. All values have been normalized to the uninfected and untreated control (left column). Darker shades of blue indicate a higher and darker shades of red indicate a lower n-fold of the respective metabolite compared to the control. Black indicates increases higher than 5-fold compared to the control. Depicted are the means of three independent experiments with three biological replicates per condition and experiment. Statistical significances were determined via ordinary two-way ANOVA and Dunnett’s correction, comparing all samples to their respective uninfected and untreated control. The n-folds and p-values are presented in **S1 Table**.

Given the fact that viral infections affect the cellular metabolism and after revealing that the IAV life cycle is mainly impaired on the level of vRNA synthesis by glycolytic interference, we wanted to get a more comprehensive understanding of metabolic alterations induced by the virus and by a treatment with 2-DG. As we know from the literature [7–9], an IAV infection has profound impacts on the host’s metabolism which especially applies to the glucose metabolism. Since IAV upregulates the glucose metabolism and 2-DG inhibits glycolysis, we expected a (partial) reversion of virus-induced metabolic changes through the inhibitor. Moreover, we were interested in metabolic changes aside from glycolysis. Via hydrophilic interaction liquid chromatography (HILIC) coupled to tandem mass spectrometry (MS/MS), as described previously [38], we analyzed major alterations of the metabolic profile of A549 cells, induced by IAV infection and/or the treatment with 2-DG after 8 h (**Fig 6**).

In accordance with the literature [7,8,10], the levels of glucose and many of the detected glycolysis intermediates were increased in infected cells, pointing towards an increase of the uptake of glucose and the rate of glycolytic activity. When 2-DG was applied, most glycolytic intermediates were detected at decreased concentrations in both, infected and uninfected cells. Counterintuitively, the amount of lactate was decreased in infected cells, which may be explained by an increased efflux upon infection [7,8] or its metabolization into other intermediates. Independent of an infection, the treatment with 2-DG clearly decreased intracellular lactate. Altogether our data confirmed a virus-mediated upregulation of glycolysis as well as its downregulation in the presence of 2-DG. In combination with our previous data this strengthens the position of metabolic inhibitors as effective antivirals by counteracting virus-induced alterations of the host metabolism.

Other metabolic pathways which are closely connected to glycolysis, such as the PPP or the TCA cycle, revealed some fascinating changes induced by 2-DG treatment or an IAV infection. 6-phosphogluconate (6-PG) exhibited an increase upon infection and supplementation of 2-DG in uninfected and infected cells. This suggested a strong redirection of glucose-6-phosphate (G-6-P) towards the PPP which was probably actively induced by the virus or by the inhibition of GPI by 2-DG. It seems that the oxidative branch of the PPP and thus the direct oxidation of glucose is upregulated upon IAV infection. Similar results have been obtained previously in chicken embryo cells [10].

Most of the detected TCA cycle intermediates decreased upon inhibition of glycolysis (abolishment of the anaplerotic function of glycolysis). The concentration of acetyl coenzyme A (acetyl-CoA), the linking intermediate between glycolysis and the TCA cycle, was increased in the presence of 2-DG and especially after an infection. Apparently, IAV infections promote the production of the important coenzyme.

Among amino acids we observed that most of them were barely affected by an infection. 2-DG led to a decrease of approximately half of the analyzed amino acids, independent of an infection. Besides, we noticed that ketogenic or partly ketogenic amino acids were barely or not reduced by 2-DG. Ketogenic amino acids can be catabolized into keto bodies (mostly TCA cycle intermediates such as acetyl-CoA, succinyl-CoA, or fumarate). Amino acids with more severely reduced concentrations after 2-DG treatment all belonged to the group of glucogenic amino acids, which means they can be catabolized into glucose through gluconeogenesis. In favor of this, we also found slightly increased concentrations of pyridoxine (vitamin B6), which is a co-factor for transaminase reactions which convert amino acids into substrates for gluconeogenesis [39,40]. The inhibition of glycolysis by 2-DG feigned the deprivation of glucose and hence mimicked starvation. Probably this triggered cells to catabolize more glucogenic amino acids.

Furthermore, we observed a disturbance of the glutathione equilibrium, one of the most important antioxidant factors for cellular redox homeostasis. In line with this finding, the disruption of glutathione and consequentially the redox homeostasis, as an important factor for IAV pathogenicity, was described before [41–43].

The effect of an IAV infection and of 2-DG on many nucleobase-related metabolites (e.g., nucleobases, nucleosides and coenzymes with related structures) was rather mild. Despite the virus-mediated increase in glycolysis, just like Ritter *et al*. reported [8], we observed no significant alteration of ATP levels 8 hpi. Even though to a mild extent, the treatment with 2-DG had the expected effect on intracellular ATP levels: 2-DG led to an ATP decrease via inhibition of glycolysis (which even consumes ATP upstream of the inhibition of GPI through the ATP-driven phosphorylation of 2-DG to 2-DG-6-P). The increase of adenosine monophosphate (AMP) in the presence of 2-DG is supported by previous publications reporting of the activation of AMP-activated protein kinase (AMPK) after glycolytic inhibition, which is triggered by a low ATP/AMP ratio [22]. Adenosine displayed the strongest increase after treatment with 2-DG among all detected metabolites. Redox-sensitive co-factors, like the different forms of nicotinamide adenine dinucleotide (here NAD^+^, NADH and NADP^+^), remained unaffected by an infection but were slightly decreased when 2-DG was applied.

Among miscellaneous metabolites two striking metabolites were creatine and phosphocreatine which were heavily reduced in the presence of 2-DG. A main task of these molecules is the conversion of ADP into ATP to sustain energy levels. The strong downregulation of creatine and phosphocreatine might have correlated with the conspicuously mild impact of IAV and 2-DG on ATP concentrations by depleting creatine/phosphocreatine pools in order to maintain sufficient ATP levels.

All described measurements so far aimed to better understand IAV and 2-DG-induced metabolic alterations. However, beside these effects, we also analyzed samples which were additionally supplied with mannose, a C2 epimer of glucose. Since mannose can be converted into fructose-6-phosphate (F-6-P) it should be able to bypass the inhibition by 2-DG to refuel glycolysis. Hence, we expected mannose to reverse some 2-DG-induced effects. Importantly, we observed this reversion, sometimes even followed by an increase, for several glycolytic intermediates (e.g., F-6-P and 1,3-bisphosphoglycerate) which indicated the antagonistic effect of mannose against glycolytic inhibition by 2-DG. F-6-P demonstrated the effects of 2-DG and mannose perfectly. Since 2-DG inhibits glycolysis directly before the conversion of G-6-P into F-6-P, this led to a decrease of it. However, the addition of mannose, which can be converted into F-6-P, strongly increased F-6-P values in uninfected and infected cells. Especially in uninfected cells the 2-DG-mediated changes of various PPP, TCA cycle and nucleobase-related intermediates were partially reversed by mannose, too. However, mannose did not always reverse up-/downregulations of metabolites mediated by 2-DG. Altogether, it seemed that the most pronounced reversions of 2-DG-mediated alterations on the metabolism by mannose took place among intermediates of the glucose metabolism and the PPP. Even though this occasionally differed between uninfected and infected cells. Nevertheless, the supplementation of mannose sometimes seemed to affect metabolites in a way which was independent of reversing 2-DG-mediated alterations.

Additionally, the same analysis was performed after an SC35M infection at an MOI of 0.1 and metabolic quenching 24 hpi (**S13 Fig**). Even though the trend of alterations induced by infection, 2-DG or mannose was similar for many metabolites compared to the 8 h setting, some metabolites displayed distinct patterns (e.g., NADP^+^, xanthine and carnitine). In general, stronger alterations were observed after an incubation period of 24 h (e.g., 6-PG, acetyl-CoA and most amino acids). However, the longer the incubation the stronger may have been the influence of other processes such as proliferation leading to altered metabolic concentrations.

Taken together, these data showed how diversely metabolic pathways are modified during IAV infections and that even metabolites from the same pathway may be affected in different manners. Furthermore, the complex connectivity between pathways or single metabolites became obvious once again. In the context of IAV infections it additionally suggested the potential of glycolytic interference to counteract IAV-induced metabolic changes as well as a function for mannose to regulate 2-DG-mediated effects.

### 2.6 Mannose circumvents the virus-restricting effect of 2-DG by refueling glycolysis

**Fig 7:**
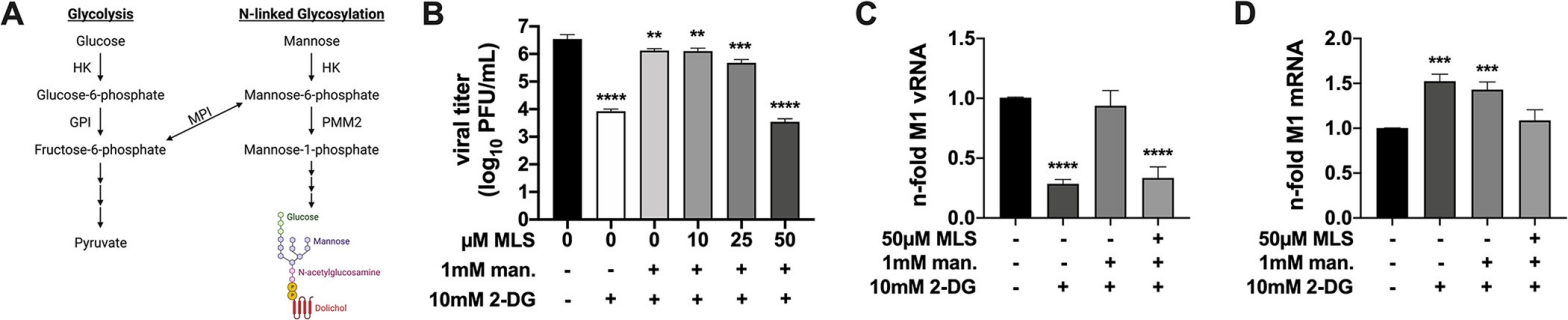
Mannose counteracts 2-DG by refueling glycolysis. **(A)** The metabolic pathways of glycolysis and N-linked glycosylation are closely connected via mannose-6-phosphate isomerase (MPI). Other enzymes depicted here are hexokinase (HK), glucose-6-phosphate isomerase (GPI), and phosphomannomutase 2 (PMM2). **(B-D)** 24 h after seeding, A549 cells were infected with SC35M at an MOI of **(B)** 0.001 or **(C+D)** 5 for 30 min and were incubated with 25 mM glucose and the indicated concentrations of 2-DG, mannose, and the MPI inhibitor MLS0315771 (MLS) for a total of **(B)** 24 h or **(C+D)** 8 h. Subsequently, **(B)** supernatants were collected to determine viral titers via plaque assay or **(C+D)** cells were lysed, their RNA isolated and cDNA synthesized using either **(C)** fluA uni12 primers to transcribe vRNA or **(D)** oligo(dT) primers to transcribe mRNA. Real-time qPCR was performed with two technical replicates per sample and values of treated samples were normalized to the untreated control. In case of mRNA detection all results were additionally normalized to a GAPDH control. **(B-D)** Depicted are the means *±* SD of three independent experiments with three biological replicates per condition and experiment. Statistical significances were determined via unpaired one-way ANOVA and Dunnett’s correction, comparing all treated samples to the untreated control. p-values are indicated as follows: < 0.05 = *, < 0.01 = **, < 0.001 = ***, < 0.0001 = ****.

As described before, glycolysis is closely linked to various other metabolic pathways and its level of activity, as seen in **Fig 6**, can have a strong impact on the abundance of other metabolites. As shown in **Fig 7A** a very close connection exists to the mannose metabolism since F-6-P from glycolysis and mannose-6-phosphate can be converted into each other by the enzyme mannose-6-phosphate isomerase (MPI). Therefore, glucose and mannose should be able to substitute each other for many of their purposes inside a cell, which would also explain some results of the metabolomic data (**Figs 6 and S13**). Indeed, the vast majority of mannose is usually shunted to glycolysis to be catabolized. The remaining mannose is mainly utilized for *N*-linked glycosylation [44]. Due to the close connection of glycolysis and *N*-linked glycosylation and since others reported that the antiviral effect of 2-DG originated from the impairment of *N*-linked glycosylation [25,45] rather than glycolytic inhibition, we aimed to dissect the interplay of these two hexoses in the context of IAV infections and the virus-restricting effects of 2-DG. Since previous publications have shown that 2-DG reduced IAV glycoprotein synthesis [24,46] and that in general the inhibition of glycosylation by 2-DG could be reversed by low doses of mannose [16,47], we supplied 2-DG-treated cells with mannose to see if this would reverse the inhibition of viral growth in our cell culture model as well (**S14A Fig**). Indeed, low concentrations of mannose restored viral titers almost completely. We observed this abolishment of the inhibitory function of 2-DG until a 1:10 ratio between mannose (1 mM) and 2-DG (10 mM). To elucidate if the reversal of inhibition can be attributed to mannose being catabolized via glycolysis or being utilized for *N*-linked glycosylation we used the MPI inhibitor MLS0315771 (MLS) to disrupt the link between these two pathways [48]. First, we determined a safe dosage of the inhibitor including potential effects on cell growth, glycolysis, and the formation of infective viral particles. We observed no significant effect on cell proliferation and cell viability but an increase of lactate in the medium in the presence of 50 μM MLS, indicating the safe use of the indicated concentrations and a higher glycolytic rate when the inhibitor is applied (**S14B and S14D Fig**). The latter can be explained by the fact that MLS prevents the redirection of F-6-P to *N*-linked glycosylation. Therefore, more glucose will be catabolized into lactate via glycolysis. Besides, we observed no significant effect on the production of viral particles (**S14E Fig**). Subsequently, we applied MLS to infected cells which were also treated with 2-DG and mannose (**Fig 7B**). We saw the typical reduction of viral titers when 2-DG alone was applied and the restoration of titers via the addition of mannose. Increasing concentrations of MLS decreased viral titers back to the level of 2-DG-treated samples which suggested that mannose restored IAV propagation mainly by driving glycolysis and not *N*-linked glycosylation. Furthermore, it also confirmed that the inhibition of glycolysis was indeed the primary antiviral mode of action of 2-DG. This got substantiated by the fact that the addition of pyruvate, the final product of glycolysis under physiological conditions, partially restored viral titers after inhibition by 2-DG (**S14F Fig**). To finally confirm the concept of the glycolytic rate as a determinant of IAV replication, we examined the effects of 2-DG, mannose, and MLS on the RNA levels of IAV after a single replication cycle of 8 h. The pattern of M1 vRNA accumulation (**Fig 7C**) strikingly resembled the pattern of viral titers (**Fig 7B**). The treatment with 2-DG led to a highly significant reduction of vRNA which was almost completely restored to the control value by supplementation of mannose. The additional administration of MLS, however, decreased the vRNA value to a similar extent as 2-DG alone did. Regarding viral mRNA accumulation (**Fig 7D**), we observed the typical slight increase after treatment with 2-DG, but barely a return to the control value when mannose was added as well. This only happened when also MLS was supplemented. To support our findings we additionally tested if either an infection or the treatment with 2-DG affected the expression of MPI and whether the mannose-mediated rescue of viral titers and RNA could also be observed for viral proteins. Via western blot we could demonstrate that MPI expression remained equal between differently treated samples (**S15A and S15B Fig**) and that viral protein accumulation indeed was restored when mannose circumvented the inhibitory effect of 2-DG (**S15A, S15C and S15D Fig**). Therefore, the restoration of viral protein accumulation via mannose possibly contributed to the overall restoration of viral replication.

Summarizing, these data corroborated that the antiviral activity of 2-DG mainly derived from a strong impairment of the synthesis of viral genomic RNA by reducing the glycolytic rate of infected host cells. Moreover, by directly or indirectly inhibiting or fueling glycolysis we found a way to turn viral reproduction on and off to a certain degree.

## 3. Discussion

Understanding the diverse interplay between the host cell metabolism and viral intruders is of importance since it may create potential new strategies to counteract viral infections. In our study we were able to improve our comprehension of metabolic virus-host interactions as well as the mode of action of glycolytic interference on the life cycle of IAV. We observed profound changes of the whole metabolic profile of infected cells (**Figs 6 and S13**), including especially upregulated amounts of many intermediates of glycolysis. By applying 2-DG, a potent inhibitor of glycolysis, many virus-induced metabolic alterations could be reversed which indicated the inhibitor’s counteraction against viral manipulations of the host. Furthermore, we showed the severe impact of glycolytic interference by 2-DG on the propagation of IAV *in vitro* (**Figs 1A and S1A**). The reduction of virus titers reached up to 4.5 orders of magnitude and hence was similar or even exceeded the effectivity of other antiviral compounds [49,50].

During the search for the point of interference within the viral life cycle we deduced that viral protein synthesis played a critical but not the sole role, because viral protein accumulation was–depending on the protein and 2-DG concentration–rather moderately affected within one replication cycle (**Fig 2F**). Interestingly, we did not at all observe a decrease in cellular protein expression after a 2-DG treatment, shown by steady signals of various cellular proteins in western blots and via *Renilla* luciferase reporter assay (**S5 Fig**). This may be indicative of a more selective effect which rather applies to viral than cellular protein translation. According to the data of **Figs 4** and **5** we assume that the predominant mechanism which is responsible for the strong reduction of IAV multiplication is a 2-DG-mediated interference with the dynamic regulation that switches the viral polymerase from a transcriptase to a replicase. Even though one hypothesis proposed that viral transcription and replication are stochastic without a switch mechanism [37] many studies suggest the opposite. The switch process of the polymerase is still not fully understood and is probably a multifactorial process determined by several viral and host factors (summarized in [31]). NP seems to be a factor in this context and was shown to have stimulatory functions on viral polymerase activity via a direct interaction with it [51–54]. However, its formerly postulated role as the potential sole regulator of transcription and replication has been refuted [55,56]. Additionally, NS1 and nuclear export protein (NEP, also known as NS2) are presumably implicated in viral replication [57–59]. Furthermore, small viral RNAs (svRNAs), which resemble the 5’ end of vRNAs, have been linked to the regulation of viral replication [60–63]. It’s been hypothesized that the role of svRNAs in viral replication is the association with a second and trans-acting polymerase which binds the 5’ end of newly synthesized vRNA [31]. Even though it once was postulated that host factors are not required to initiate viral replication [53], many candidates that can associate with vRNP components [31,54,64–67] and thereby potentially influence the process, such as the recently described acidic nuclear phosphoprotein 32 (ANP32) [68–70], have been identified. Since the nuclear matrix and chromatin of infected cells were postulated to constitute a platform for viral transcription and replication [71–73], various potential host factors are associated with these sub-nuclear structures [74–77]. Linking the described regulators of IAV polymerase activity and the here presented data, it is quite possible that metabolic interference via 2-DG impairs the IAV replication-associated function or the expression of one or several of these viral or host factors. After all we know, however, it is also possible that there is no strong switching mechanism controlling viral transcription or replication. Potentially the abundance of both processes is basically stochastic but can be modulated in favor of transcription or replication in a time-dependent manner. Combining the insights from previous publications with our data it is imaginable that the antiviral effect of 2-DG operates in several steps. One scenario could be that inhibition by 2-DG leads to a primary antiviral effect by interfering with the function of the initial transcription and replication complexes which could explain the generalized lower levels of mRNA and vRNA until 7 hpi (**Fig 4**). A secondary effect could be the seemingly selective impairment of the accumulation of viral proteins (**Figs 2 and S5**). A possible explanation for this selective effect could be events mediated by some viral proteins. NS1, for example, has the ability to initiate viral translation by recruiting ribosomes to viral mRNAs [78]. In our experiments, a mild reduction in the NS1 expression might be enough to affect viral translation initiation. Furthermore, NS1 is involved in host gene shutoff by inhibiting polyadenylation of cellular transcripts [79], thus causing their degradation. Besides, the reduction of PA could further reduce host gene shutoff, since PA is necessary for cap-snatching [80] and PA-X degrades cellular transcripts [81]. Reduced host gene shutoff increases the competition between viral and cellular mRNA for translation and hence reduces viral protein accumulation. Consequently, a lack of nascent polymerase complexes may have a stronger impact on replication than transcription since replication requires a second polymerase for the binding of nascent cRNA and vRNA strands. Possibly the 2-DG-mediated reduction of viral protein accumulation is an inhibitory step that is preceding and, to a certain extent, causing the impairment of viral replication. This idea is substantiated by the mannose-mediated simultaneous rescue of viral protein expression, viral genome replication and viral growth in 2-DG-treated cells (**Figs 7 and S15**). Alternatively or additionally, treatment with 2-DG might impair the synthesis of any of the afore-mentioned modulators of the viral polymerase which may contribute to the clear reduction of replication and the prolonged phase of transcription which could be observed in some cell types. Of course, the variety of potential 2-DG-mediated influences on viral replication is huge and on top of that we cannot fully exclude an off-target interaction which may play a role here. However, the latter seems highly unlikely based on the data we generated through the supplementation of mannose and MLS in the presence of 2-DG (**Fig 7**). It will be interesting to examine if and how severely 2-DG influences the expression or interactions of the afore-mentioned viral and cellular factors with the complex replication machinery of IAV.

Furthermore, our data suggest that the predominant antiviral mode of action of 2-DG is the inhibition of glycolysis. Decades ago it has been postulated that the impairment of *N*-linked glycosylation is responsible for the antiviral effect of 2-DG [45]. The fact that inhibition of the enzyme MPI, which links glycolysis and glycosylation, abolished the restoration of viral titers and vRNA levels by mannose after treatment with 2-DG (**Fig 7B and 7C**) lets us oppose this view. Our data indicate that the positive effect of mannose on IAV replication mainly (but not necessarily exclusively) derives from fueling glycolysis via its conversion into F-6-P by MPI. Moreover, the partial restoration of viral titers by the supplementation of pyruvate after inhibiting glycolysis substantiates the assumption that glycolysis and its intermediates are crucial for virus reproduction. Probably the availability of glycolytic intermediates, which are needed to fuel other pathways and to synthesize macromolecules such as nucleotides and amino acids, is the most critical factor. Extrapolations predicted only a very minor extra demand for energy (~1% of the total energetic budget of a eukaryotic cell) to synthesize viral progeny during the characteristic time of an influenza infection [82]. Therefore, we assume that a potential role of ATP in viral replication may rather not be its availability for synthesis reactions.

As reviewed previously [83], 2-DG has various direct and indirect mechanism by which it can negatively affect normal cellular functions (e.g., inhibition of glycolysis and glycosylation or induction of AMPK and UPR). Therefore, a certain cytotoxicity–which heavily depends on the dosage, type of administration and the type of cell, tissue, or organism–must be considered. However, we could demonstrate the tolerability and the quickness of effectivity of the antimetabolite in immortalized and primary cells (**Figs 1C–1F, S1B, S4B and S4C**). Our *in vitro* data and previous reports [26,27] support the performance of more *in vivo* studies and clinical trials to assess the safety of 2-DG and its efficiency to treat virus infections in model organisms or even humans. Several such studies have already reported the safety of 2-DG in animal models in the context of other virus infections [84] or different fields of research [85–87], especially when administered in continuous low doses. This could even be confirmed in clinical trials [88,89]. Very recent phase II and III clinical trials in India [90,91] demonstrated the safety and effectiveness of 2-DG when applied in addition to the standard of care to treat severe COVID-19 patients. As studies in which a virus infection was more successfully treated in humans through metabolic interference, these clinical trials may become a milestone in the development of host-targeted metabolic drugs as antivirals. However, some studies [92,93] and its poor pharmacokinetic properties, e.g., its short plasma half-life [94], suggest that 2-DG itself may never become a licensed drug. Nevertheless, it is a useful tool to examine the principles of glycolytic interference and novel 2-DG analogs or other glycolytic inhibitors possibly boast a better pharmacological suitability [95]. We discussed the influence of 2-DG and other metabolic inhibitors on different respiratory viruses before [6]. 2-DG proved to have a broad antiviral activity against various single-stranded RNA viruses which replicate either in the nucleus or in the cytoplasm. Interestingly, adenoviruses, which are double-stranded DNA viruses, even benefit from glycolytic inhibition. These differences might derive from different replication strategies and alternative metabolic interference strategies might be more suitable for certain types of viruses depending on their genome and site of replication. Since dependence on the host metabolism is a universal feature of all viruses, differential and strictly determined metabolic treatments may be able to alleviate all types of virus infections in the future. However, before this may become reality, we need to gain a more comprehensive understanding of metabolism-related virus-host interactions, including virus-induced metabolic modifications, specific metabolic needs of different viruses and how exactly metabolic treatments affect the viral life cycle as well as the host. We are positive that this specific field of research deserves more attention to elaborate metabolic interference and make it become a realistic and sensible treatment option in the future.

## 4. Materials and methods

### 4.1 Ethics statement

All donors of human lung explants gave their written content to donate lung tissue for scientific purposes. Ethical approval was given by the Deutsche Ärztekammer (AZ: 2016-265-f-S).

### 4.2 Cell lines and viruses

Human adenocarcinomic alveolar basal epithelial cells (A549, American type culture collection (ATCC), CCL-185), human embryonic kidney (HEK) 293t cells (ATCC, CRL-3216) and human adenocarcinomic lung epithelial Calu-3 cells (ATCC, HTB-55) were cultured in the high glucose variant of Dulbecco’s modified Eagle’s medium (DMEM, Sigma-Aldrich, D5796) supplemented with 10% fetal bovine serum (FBS). Madin-Darby canine kidney (MDCK) II cells (Institute of Virology, WWU Muenster, Germany) were cultured in minimum essential medium (MEM, Sigma-Aldrich, M4655) supplemented with 10% fetal bovine serum (FBS). The primary cells human bronchial epithelial cell (HBEpC, PromoCell, C-12640) were cultured in airway epithelial cell growth medium (AECGM, PromoCell, C-21060). Tumor-free human lung explants were obtained from various donors right after surgery at the University Hospital Muenster and were cultured in Roswell Park Memorial Institute-1640 medium (RPMI-1640, Sigma-Aldrich, R8758) supplemented with 100 U/mL penicillin and 0.1 mg/mL streptomycin. The donors gave written consent for the tissue to be used for scientific purposes. All cells were kept at 37°C and 5% CO_2_. Mouse-adapted A/Seal/Massachusetts/1/80 H7N7 (SC35M), A/Panama/2007/1999 H3N2 (Pan/99) and A/WSN/1933 H1N1 (WSN) are recombinant influenza A virus (IAV) strains which were propagated in MDCK II cells.

### 4.3 Infection and treatment

Viruses were diluted to the desired multiplicity of infection (MOI) in phosphate-buffered saline (PBS) supplemented with 0.2% bovine serum albumin (BSA), 1 mM MgCl_2_, 0.9 mM CaCl_2_, 100 U/mL penicillin and 0.1 mg/mL streptomycin. Cells were washed once with PBS and incubated for 30 min at 37°C and 5% CO_2_ with the respective amount of virus. Afterwards A549, HEK293T and Calu-3 cells were washed once more with PBS and then incubated for the depicted periods in DMEM (Thermo Fisher Scientific, A14430) containing 0.2% bovine serum albumin (BSA), 100 U/mL penicillin and 0.1 mg/mL streptomycin, 25 mM D-glucose, 2 mM L-glutamine and the respective concentration of inhibitor/supplement. The medium did not contain sodium pyruvate, HEPES and phenol red. HBEpCs were washed once with PBS after an infection and incubated in AECGM, containing the respective amounts of inhibitor/supplement for the depicted periods of the experiments. Human lung explants (~100 mg) were infected with 2 x 10^5^ infectious virus particles as described previously [96], but without any interferon or bafilomycin. After washing the tissue 1 hpi, it was incubated in fresh RPMI supplemented with 2 mM L-glutamine, 100 U/mL penicillin, 0.1 mg/mL streptomycin, 0.1% bovine serum albumin and the indicated concentrations of inhibitor. 2-deoxy-d-glucose (2-DG, Sigma-Aldrich, D8375), d-(+)-mannose (Sigma-Aldrich, M6020) and sodium pyruvate (Sigma-Aldrich, P5280) were dissolved in H_2_O to 1 M (2-DG and mannose) and 2 M (sodium pyruvate) stock solutions. In the case of infections with Pan/99 the assay medium additionally contained TPCK-treated trypsin (1:4000). MLS0315771 (MedChemExpress, HY-112945) was dissolved in dimethyl sulfoxide (DMSO) to a stock concentration of 10 mM. Actinomycin D (Roth, 8969.1) was dissolved in DMSO to a stock concentration of 1 mg/mL and was applied 6 hpi at a final concentration of 10 μg/mL for the indicated durations. MG132 (MedChemExpress, 133407-82-6) was dissolved in DMSO to a stock concentration of 10 mM and was applied 2 hpi at a final concentration of 20 μM for 6 h. For the stimulation of immune responses via RNA transfection, RNA was isolated from mock-infected and SC35M-infected (MOI of 5) cells 8 hpi, as described in **4.8**. 100 ng RNA per well was transfected using HiPerFect Transfection Reagent (QIAGEN) according to the manufacturer’s protocol for 6 h in the presence of the depicted inhibitor concentrations.

### 4.4 Plaque titration

After the indicated periods of infection, the supernatants were collected and used to determine the number of infectious virus particles. Confluent MDCK II cells were infected with serial dilutions of the supernatants in PBS containing 0.2% bovine serum albumin (BSA), 1 mM MgCl_2_, 0.9 mM CaCl_2_, 100 U/mL penicillin and 0.1 mg/mL streptomycin for 30 min at 37°C and 5% CO_2_. Subsequently the supernatants were replaced with MEM/BA containing 0.21% BSA, 0.21% NaHCO_3_, 1 mM MgCl_2_, 0.01% DEAE-dextran, 0.9 mM CaCl_2_, 100 U/ml penicillin, 0.1 mg/ml streptomycin and 0.9% purified agar. After an incubation for 2–3 days at 37°C and 5% CO_2_ the overlay was removed and cells were stained with a Coomassie staining solution (45% ddH_2_O (v/v), 45% methanol (v/v), 10% acetic acid (v/v) and 0.25% Coomassie Brilliant blue R-250 (w/v)). Cell free plaques in the monolayer were counted as plaque-forming units per milliliter (PFU/mL).

### 4.5 Cytotoxicity assays

Potential cytotoxic effects of inhibitors were assessed by three different methods: lactate dehydrogenase (LDH) assay, trypan blue staining and flow cytometry. LDH assays were performed with the CytoSelect LDH cytotoxicity assay kit (Bio Cat, CBA-241-CB) according to the manufacturer’s manual. Trypan blue exclusion was done by mixing a 0.4% trypan blue dye (Invitrogen) 1:1 with a sample’s cell suspension and having the automated cell counting machine Countess II (Invitrogen) determine the number of living cells. Determination of living cells via flow cytometry is described below in section **4.11**.

### 4.6 Glycolytic rate test

The induced assay version of the glycolytic rate test (Agilent, Kit 103344–100) was performed with a Seahorse XFe96 Analyzer (Agilent) according to the manufacturer’s instructions. The assay medium was supplemented with 25 mM D-glucose and 2 mM L-glutamine to match other experimental conditions. Concomitantly, the final injection of 2-DG was set to 125 mM. After three measured points to obtain the basal glycolytic level, the indicated concentrations of inhibitor were injected and the glycolytic rate was measured for 1 h before continuing with the standard procedure.

### 4.7 Lactate assay

To determine the concentration of lactate in the supernatants of samples and thus have an indirect assay to assess glycolytic activity, the L-Lactate Assay Kit II (PK-CA577-K607) from PromoCell was used according to the manufacturer’s instruction.

### 4.8 Reverse transcription and quantitative real-time PCR

At the end of an infection and/or treatment period, total RNA was isolated using the RNeasy Plus Mini Kit (Qiagen). The procedure was done according to the manufacturer’s manual. Reverse transcription was performed with the RevertAid H Minus Reverse Transcriptase (Thermo Fisher Scientific) and oligo(dT) primers (Eurofins Genomics) for detection of mRNA or a fluA uni12 forward primer [97] (Sigma-Aldrich, 5’-AGCAAAAGCAGG-3’) to detect vRNA according to the manufacturer’s protocol. The obtained cDNA was used for real-time qPCR with a LightCycler 480 II (Roche) and Brilliant III SYBR Green (Agilent) according to the manufacturer’s instructions. The following primers were used during qPCR: influenza matrix protein M1 forward (5’-AGA TGA GTC TTC TAA CCG AGG TCG-3’) and reverse (5’-TGC AAA AAC ATC TTC AAG TCT CTG-3’), IL-6 forward (5’-AGA GGC ACT GGC AGA AAA CAA C-3’) and reverse (5’-AGG CAA GTC TCC TCA TTG AAT CC-3’), CXCL8 forward (5’-ACT GAG AGT GAT TGA GAG TGG AC-3’) and reverse (5’-AAC CCT CTG CAC CCA GTT TTC-3’), DDX58 forward (5’-CCT ACC TAC ATC CTG AGC TAC AT-3’) and reverse (5’-TCT AGG GCA TCC AAA AAG CCA-3’), MxA forward (5’-GTT TCC GAA GTG GAC ATC GCA-3’) and reverse (5’-GAA GGG CAA CTC CTG ACA GT-3’) and human glyceraldehyde 3-phosphate dehydrogenase (GAPDH) forward (5’-GCA AAT TCC ATG GCA CCG T-3’) and reverse (5’-GCC CCA CTT GAT TTT GGA GG-3’). GAPDH, as a housekeeping gene, was used for the normalization of PCR results. The relative n-fold was calculated using the 2^-ΔΔCT^ method [98].

### 4.9 Strand-specific quantitative real-time RT-PCR

Total RNA was isolate as described in **4.8**. Reverse transcription was performed by using Maxima Reverse Transcriptase (Thermo Fisher Scientific) according to the manufacturer’s instructions and specific primers (Eurofins Genomics) for the different types of RNA as reported previously [33]. Primers for SC35M targets were designed according to the sequences DQ266097, DQ226096 and DQ266095 (Influenza Research Database) while WSN primer sequences were obtained from a previous publication [33].

### 4.10 Western blot

Samples were lysed at 4°C with radioimmunoprecipitation assay (RIPA) buffer (25 mM Tris-HCl pH 8, 137 mM NaCl, 10% glycerol, 0.1% SDS, 0.5% NaDOC, 1% NP-40, 2 mM EDTA pH 8, 200 μM Pefabloc, 5 μg/mL aprotinin, 5 μg/mL leupeptin, 1 mM sodium orthovanadate and 5 mM benzamidine). Cell debris was removed via centrifugation and protein concentrations were determined by Bradford assay. Samples were adjusted to the same protein concentration, mixed with the appropriate amount of Laemmli sample buffer and then proteins were separated and visualized by sodium dodecyl sulfate polyacrylamide gel electrophoresis (SDS-PAGE) and western blot analysis. The following primary antibodies were used to detect their respective proteins: ERK2 (rabbit, polyclonal, Santa Cruz, sc-154), α-tubulin (mouse, monoclonal, Sigma-Aldrich, T6199), β-actin (mouse, monoclonal, Santa Cruz, sc-47778), MPI (rabbit, polyclonal, GeneTex, GTX103682), M1 (mouse, monoclonal, Biorad, MCA401), NP (rabbit, polyclonal, GeneTex, GTX125989), NS1 (rabbit, polyclonal, GeneTex, GTX125990), and PA (rabbit, polyclonal, GeneTex, GTX125932). ERK2 served as the loading control for whole cell lysates. Fluorescence signals were visualized by using fluorophore-labelled secondary antibodies: IRDye 680RD Donkey anti-Mouse (LI-COR, 926–68072), IRDye 680RD Donkey anti-Rabbit (LI-COR, 926–68073), IRDye 800CW Donkey anti-Mouse (LI-COR, 926–32212), and IRDye 800CW Donkey anti-Rabbit (LI-COR, 926–32213). Images were taken with the ODYSSEY F_C_ Imaging System (LI-COR).

### 4.11 Flow cytometry

At the end of an infection with or without treatment, cells were trypsinized and subsequently stained for analysis via flow cytometry with the FACSCalibur (Becton Dickinson) flow cytometer. At first, cells were stained with eBioscience Fixable Viability Dye eFluor 660 (Invitrogen, 65-0866-14) for 30 min at 4°C in the dark. Afterwards the samples were fixated and permeabilized for 20 min and 60 min at 4°C in the dark using BD Cytofix/Cytoperm solution and BD Perm/Wash solution (BD Biosciences), respectively. Intracellular staining of influenza A nucleoprotein was done by applying the anti influenza A (nucleoprotein)–FITC antibody (OriGene, AM00924FC-N) for 60 min at 4°C in the dark. FlowJo software v10 (Becton Dickinson) was used to analyze the data obtained by flow cytometry. 10^5^ cells of each sample were analyzed. The gating strategy is displayed in **S16 Fig**.

### 4.12 Minigenome assay

Using Lipofectamine 2000 (Invitrogen), HEK293T cells were transfected with polymerase II-driven pCAGGS plasmids coding for PA, PB1, PB2 and NP of SC35M as well as the pTK-Renilla plasmid coding for the transfection control *Renilla* luciferase. Alternatively, polymerase II-driven pCAGGS plasmids coding for NP, PA, PB1 and PB2 or a replication-competent but transcription-deficient PB2 mutant (PB2 R+/T-), PB2-361A [35,36]), of WSN were transfected. An additional plasmid was one of two polymerase I-driven pUC18 plasmids encoding either a vRNA-like or cRNA-like *Firefly* luciferase template. 4 h post transfection the medium was replaced with DMEM (Thermo Fisher Scientific, A14430) containing 0.2% BSA, 100 U/mL penicillin and 0.1 mg/mL streptomycin, 25 mM D-glucose, 2 mM L-glutamine and the respective concentration of 2-DG. 24 h post transfection total RNA was isolated for subsequent PCR analyses as described in **4.8** and **4.9** or the Dual-Luciferase Reporter Assay System (Promega) was used according to the manufacturer’s manual. For measurements of relative light units (RLU) the luminometer MicroLumat*Plus* LB 96V (Berthold Technologies) and the software WinGlow (Berthold Technologies) were used. Plasmids were generated as described previously [99].

### 4.13 RNP durability assay

HEK293T cells were transfected with pCAGGS plasmids coding for PA, PB1, PB2 and NP of SC35M or PA, PB1, PB2, NP and a catalytically inactive version of PB1 (PB1(-)), PB1-D445A/D446A [37], of WSN using Lipofectamine 2000 (Invitrogen). 24 h post transfection cells were infected with SC35M or WSN at an MOI of 5 (see **4.3**) and incubated with or without cycloheximide (100 μg/mL) and various concentrations of 2-DG. 6 hpi cell lysates were taken and subjected to strand-specific quantitative real-time RT-PCR (see **4.9**).

### 4.14 Metabolic profiling by HILIC-MS/MS

24 h after seeding 1.5 x 10^6^ A549 cells in 6 cm dishes, they were mock-infected or infected with SC35M at an MOI of 5/0.1. 8/24 hpi cells were washed twice with PBS and 400 μL pre-cooled (4–8°C) acetonitrile (ACN)/water (4+1, v/v) including 50 μM d-phenylglycine as internal standard was added for metabolic quenching. Until further preparation the samples were kept at 4–8°C. Cells were then detached using a sterile cell scraper. The dish was washed with additional 800 μL ACN/water (4+1, v/v) and pooled with the respective cell sample. Further preparation of samples as well as chromatographic and mass spectrometric analysis were performed as described previously [38].

## Supporting information

S1 Fig Effects of 2-DG on IAV propagation and cell growth. 24 h after seeding, A549 cells were infected with SC35M at an MOI of **(A+B)** 0.01 for 30 min or **(C+D)** remained uninfected and were incubated in the presence of the indicated concentrations of 2-DG or its solvent water for 24 h. Subsequently, cells were **(A+B)** stained with an NP antibody and a live/dead marker and were analyzed via flow cytometry or **(C+D)** were detached to assess the number of living cells as well as the viability via trypan blue exclusion in an automated cell counter. **(A-D)** Depicted are the means *±* SD of three independent experiments with three biological replicates per condition and experiment. Statistical significances were determined via **(A, C, D)** unpaired one-way ANOVA and Dunnett’s correction, comparing all treated samples to the water control or **(B)** ordinary two-way ANOVA with Dunnett’s correction, comparing all treated samples of both groups to their respective water control. p-values are indicated as follows: < 0.05 = *, < 0.01 = **, < 0.001 = ***, < 0.0001 = ****.
(TIFF)

S2 Fig Effects of 2-DG and oligomycin A on IAV propagation and cell growth. 24 h after seeding, A549 cells were infected with SC35M at an MOI of 0.001 for 30 min and were incubated in the presence of 25mM glucose and the indicated concentrations of 2-DG and/or oligomycin A or their solvents water and DMSO for 24 h. **(A)** Subsequently, supernatants were collected to determine viral titers via plaque assay and **(B+C)** cells were detached to assess the number of living cells as well as the viability via trypan blue exclusion in an automated cell counter. **(A-C)** Depicted are the means *±* SD of three independent experiments with three biological replicates per condition and experiment. Statistical significances were determined via unpaired one-way ANOVA and Tukey’s correction, comparing all samples with each other. p-values are indicated as follows: < 0.05 = *, < 0.01 = **, < 0.001 = ***, < 0.0001 = ****.
(TIFF)

S3 Fig Effects of 2-DG on the immune induction. 24 h after seeding, uninfected cells were transfected with cellular or viral RNA and treated with the indicated 2-DG concentrations for 6 h. Subsequently, cells were lysed, their RNA isolated and cDNA synthesized using oligo(dT) primers to transcribe mRNA. Real-time qPCR was performed with two technical replicates per sample and values of all other samples were normalized to the unstimulated water control. Additionally, all results were normalized to a GAPDH control. Depicted are the means *±* SD of three independent experiments with three biological replicates per condition and experiment. Statistical significances were determined via ordinary two-way ANOVA with Dunnett’s correction, comparing all treated samples of both groups to their respective water control. p-values are indicated as follows: < 0.05 = *, < 0.01 = **, < 0.001 = ***, < 0.0001 = ****.
(TIFF)

S4 Fig Effects of 2-DG on human primary cells and IAV propagation. **(A)** Human lung explants were infected with 2 x 10^5^ SC35M particles for 30 min. Afterwards they were incubated with 11.1 mM glucose and the indicated concentrations of 2-DG and supernatants were collected 1, 24 and 48 hpi to determine viral titers via plaque assay. **(B-I)** After reaching *≈* 90% confluency **(B)** uninfected HBEpCs were treated with the indicated concentrations of 2-DG or its solvent water for 24 h. Afterwards the supernatants were used to perform LDH assays to determine the relative cytotoxicity of the treatment. **(C-I)** HBEpCs were infected with SC35M at an MOI of **(C)** 1, **(D, F, H)** 0.01 or **(E, G, I)** 5 for 30 min and were incubated with 6 mM glucose and the indicated concentrations of 2-DG for a total of **(D, F, H)** 24 h or **(E, G, I)** 8 h. Subsequently, **(C-E)** supernatants were used to **(C)** perform lactate assays in order to indirectly assess the glycolytic activity and **(D+E)** determine viral titers via plaque assay. **(F-I)** Additionally, cells were lysed, their RNA isolated and cDNA synthesized using either **(F+H)** oligo(dT) primers to transcribe mRNA or **(H+I)** fluA uni12 primers to transcribe vRNA. Real-time qPCR was performed with two technical replicates per sample and values of treated samples were normalized to the water control. In case of mRNA detection, all results were additionally normalized to a GAPDH control. Depicted are the means *±* SD of three independent experiments with three biological replicates per condition and experiment. Statistical significances were determined via **(A-C)** ordinary two-way ANOVA and Dunnett’s correction, comparing each treated sample to its respective water control. **(D-I)** Other significances were determined via unpaired one-way ANOVA and Dunnett’s correction, comparing all treated samples to the water control. p-values are indicated as follows: < 0.05 = *, < 0.01 = **, < 0.001 = ***, < 0.0001 = ****.
(TIFF)

S5 Fig 2-DG does not reduce cellular protein expression. **(A)** 24 h after seeding, HEK293T cells were transfected with an empty vector or a plasmid containing the *Renilla* luciferase gene which is under the control of a constitutive herpes simplex virus thymidine kinase promoter. Subsequently, the cells were incubated with the shown 2-DG concentrations. After 24 h, cells were lysed and the n-fold of relative light units (RLU) in comparison to the water control was measured via luciferase assay. Depicted are the means ± SD of three independent experiments with three biological replicates per condition and experiment. Statistical significances were determined via unpaired one-way ANOVA and Dunnett’s correction, comparing all treated samples to the water control. **(B)** 24 h after seeding, A549 cells were infected with SC35M at an MOI of 0.001 for 30 min and were incubated with 25 mM glucose and the indicated concentrations of 2-DG for a total of 24 h. Protein lysates of triplicates were unified to yield sufficient protein amounts. Proteins were separated via SDS-PAGE. Visualization was done using primary antibodies against α-tubulin (mouse), β-actin (mouse), ERK2 (rabbit) and M1 (mouse) and fluorescence-labelled anti-mouse (donkey) and anti-rabbit (donkey) secondary antibodies. Depicted are representative protein bands from one out of three independent experiments. **(C-F)** Densitometric analyses were performed to quantify protein accumulation. The n-folds were calculated in regard to **(C-D)** the mock control or **(F)** the infected and untreated sample. Depicted are the means ± SD of three independent experiments. Statistical significances were determined via unpaired one-way ANOVA and Dunnett’s correction, comparing all other samples to **(C-D)** the mock control or **(F)** the infected and untreated sample. p-values are indicated as follows: < 0.05 = *, < 0.01 = **, < 0.001 = ***, < 0.0001 = ****.
(TIFF)

S6 Fig 2-DG does not affect viral mRNA and protein turnover. 24 h after seeding, A549 cells were infected with SC35M at an MOI of 5 for 30 min and were incubated with 25 mM glucose and the indicated concentrations of 2-DG. **(A)** 6 hpi media were replaced with the same media containing actinomycin D (10 μg/mL) and cells were incubated in it for the depicted time points. Subsequently, cells were lysed, their RNA isolated and cDNA synthesized using oligo(dT) primers. Real-time qPCR was performed with two technical replicates per sample and values of treated samples were normalized to the water control. All results were additionally normalized to a GAPDH control. Depicted are the means ± SD of three independent experiments with three biological replicates per condition and experiment. Statistical significances were determined via ordinary two-way ANOVA and Sidak’s correction, comparing the samples of a common time point with each other. **(B)** 2 hpi MG132 was added to the media (20 μM) and cells were incubated with it for another 6 h. Protein lysates of triplicates were unified to yield sufficient protein amounts. Proteins were separated via SDS-PAGE. Visualization was done using primary antibodies against PA (rabbit), M1 (mouse) and ERK2 (rabbit) and fluorescence-labelled anti-mouse (donkey) and anti-rabbit (donkey) secondary antibodies. Depicted are representative protein bands from one out of three independent experiments. **(C+D)** Densitometric analyses were performed to quantify protein accumulation by first normalizing PA and M1 to the loading control ERK2 and then normalizing all other samples to the infected but untreated sample. MG132(+) and MG132(-) samples were normalized independently. Depicted are the means ± SD of three independent experiments. Statistical significances were determined via unpaired one-way ANOVA and Dunnett’s correction, comparing all other samples to the infected but untreated sample (second/sixth lane). p-values are indicated as follows: < 0.05 = *, < 0.01 = **, < 0.001 = ***, < 0.0001 = ****.
(TIFF)

S7 Fig Effect of 2-DG on IAV transcription and replication in Calu-3 cells. 24 h after seeding, Calu-3 cells were infected with SC35M at an MOI of 5 for 30 min and were incubated with 25 mM glucose and 10 mM 2-DG or its solvent water for a total of 8 h. Subsequently, cells were lysed, their RNA isolated and cDNA synthesized using either **(A)** oligo(dT) primers, **(C)** fluA uni12 primers or **(B+D)** specific primers to transcribe mRNA or vRNA of M1 and NP. Real-time qPCR was performed with two technical replicates per sample and values of treated samples were normalized to the water control. In case of mRNA detection, all results were additionally normalized to a GAPDH control. Depicted are the means ± SD of three independent experiments with three biological replicates per condition and experiment. Statistical significances were determined via unpaired t-test with Welch’s correction. p-values are indicated as follows: < 0.05 = *, < 0.01 = **, < 0.001 = ***, < 0.0001 = ****.
(TIFF)

S8 Fig Impairment of IAV replication by 2-DG is not strain-specific. 24 h after seeding, A549 cells were infected with Pan/99 at the depicted MOIs for 30 min and were incubated with the indicated concentrations of 2-DG or its solvent water for a total of **(A-C)** 24 h or **(D+E)** 8 h. Subsequently, **(A)** supernatants were collected to determine viral titers via plaque assay or **(B-E)** cells were lysed, their RNA isolated and cDNA synthesized using either **(B+D)** oligo(dT) primers to transcribe mRNA or **(C+E)** fluA uni12 primers to transcribe vRNA. Real-time qPCR was performed with two technical replicates per sample and values of treated samples were normalized to the water control. In case of mRNA detection, all results were additionally normalized to a GAPDH control. Depicted are the means ± SD of three independent experiments with three biological replicates per condition and experiment. Statistical significances were determined via unpaired one-way ANOVA and Dunnett’s correction, comparing all treated samples to the water control. p-values are indicated as follows: < 0.05 = *, < 0.01 = **, < 0.001 = ***, < 0.0001 = ****.
(TIFF)

S9 Fig Time-dependent effects of 2-DG on IAV transcription and replication. 24 h after seeding, A549 cells were infected with SC35M at an MOI of 5 for 30 min and were incubated without or with 10 mM 2-DG in the presence of 25 mM glucose for a total of 8 h. **(A+B)** Each hour cells were lysed, their RNA isolated and cDNA synthesized using **(A)** oligo(dT) primers or **(B)** fluA uni12 primers. Real-time qPCR was performed with two technical replicates per sample. **The raw data are the same as in Fig 4A and 4B but the values of each time point were normalized to the water control of the same time point.** Depicted are the means *±* SD of three independent experiments with three biological replicates per condition and experiment. Statistical significances were determined via ordinary two-way ANOVA and Sidak’s correction, comparing the treated sample of each time point to its respective water control. p-values are indicated as follows: < 0.05 = *, < 0.01 = **, < 0.001 = ***, < 0.0001 = ****.
(TIFF)

S10 Fig The replicative capacity of the IAV polymerase is not impaired by 2-DG. 24 h after seeding, HEK293T cells were transfected with plasmids encoding NP, PA, PB1 and PB2 R+/T- of WSN as well as either a **(A)** cRNA-like or **(B)** vRNA-like template of the Firefly (FF) luciferase. The negative control was transfected with an empty vector instead of PB2 while the positive control was transfected with wild type PB2 instead of PB2 R+/T-. 4 h later the transfection solution was replaced with medium containing 25 mM glucose and the indicated concentrations of 2-DG for another 20 h. Subsequently, cells were lysed, their RNA isolated and cDNA synthesized using specific primers to transcribe **(A)** vRNA and **(B)** mRNA of FF. Real-time qPCR was performed with two technical replicates per sample and values of treated samples were normalized to the water control. Depicted are the means *±* SD of three independent experiments with three biological replicates per condition and experiment. Statistical significances were determined via unpaired one-way ANOVA and Dunnett’s correction, comparing all other samples to the **(A)** water control or **(B)** the PB2 positive control. p-values are indicated as follows: < 0.05 = *, < 0.01 = **, < 0.001 = ***, < 0.0001 = ****.
(TIFF)

S11 Fig 2-DG attenuates but prolongs mRNA synthesis and reduces cRNA and vRNA accumulation. 24 h after seeding, A549 cells were infected with SC35M at an MOI of 5 for 30 min and were afterwards incubated without or with 10 mM 2-DG in the presence of 25 mM glucose for a maximum of 8 h. Each hour cells were lysed, their RNA isolated and cDNA synthesized using specific primers to transcribe mRNA, cRNA and vRNA of the SC35M gene segments 1 (PB2), 5 (NP) and 6 (NA). Real-time qPCR was performed with two technical replicates per sample. All values were normalized to the water control 1 hpi. Depicted are the means ± SD of three independent experiments with three biological replicates per condition and experiment. Statistical significances were determined via ordinary two-way ANOVA and Sidak’s correction, comparing the treated sample of each time point to its respective water control. p-values are indicated as follows: < 0.05 = *, < 0.01 = **, < 0.001 = ***, < 0.0001 = ****.
(TIFF)

S12 Fig 2-DG does not impair IAV RNP durability. 24 h after seeding, HEK293T cells were transfected with plasmids containing **(A+B)** the SC35M sequences of PA, PB1, PB2 and NP or **(C+D)** the WSN sequences of PA, PB1 or PB1(-), PB2 and NP. 4 h later the transfection solution was replaced with fresh medium for another 20 h. Subsequently, cells were infected with SC35M at an MOI of 5 for 30 min and were incubated with the indicated concentrations of 2-DG and 100 μg/mL cycloheximide. A negative control was previously transfected with an empty vector instead of PA while a positive control was not treated with cycloheximide. 6 hpi, cells were lysed, their RNA isolated and cDNA synthesized using specific primers to transcribe cRNA and vRNA of the **(A+B)** SC35M or **(C+D)** WSN gene segment 6 (NA). Real-time qPCR was performed with two technical replicates per sample. Statistical significances were determined via unpaired one-way ANOVA and Dunnett’s correction, comparing all other samples to the respective water control. p-values are indicated as follows: < 0.05 = *, < 0.01 = **, < 0.001 = ***, < 0.0001 = ****.
(TIFF)

S13 Fig Metabolic alterations induced by IAV infection and glycolytic treatment within 24 h. A549 cells were mock-infected or infected with SC35M at an MOI of 0.1 and were subsequently incubated in DMEM (containing 25 mM glucose) with or without 10 mM 2-DG and 1 mM mannose as indicated. 24 hpi metabolic activity was quenched and intracellular metabolites were relatively quantified via HILIC-MS/MS. All values have been normalized to the uninfected and untreated control (left column). Darker shades of blue indicate a higher and darker shades of red indicate a lower n-fold of the respective metabolite compared to the control. Black indicates increases higher than 5-fold compared to the control. Depicted are the means of three independent experiments with three biological replicates per condition and experiment. Statistical significances were determined via ordinary two-way ANOVA and Dunnett’s correction, comparing all samples to their respective uninfected and untreated control. The n-folds and p-values are presented in **S2 Table**.
(TIFF)

S14 Fig Effects of mannose, MLS0315771, and pyruvate on IAV propagation and A549 cells. 24 h after seeding, A549 cells were infected with SC35M at an MOI of 0.001 or for 30 min and were incubated in the presence of the indicated concentrations of metabolites and inhibitors or their solvents for a total of 24 h. Subsequently, **(A, D-F)** supernatants were collected to determine **(A, E, F)** viral titers via plaque assay and **(D)** extracellular lactate concentrations via lactate assay or **(B+C)** cells were detached to assess the number of living cells and the viability via trypan blue exclusion and an automated cell counter. Depicted are the means *±* SD of three independent experiments with three biological replicates per condition and experiment. Statistical significances were determined via unpaired one-way ANOVA and Dunnett’s correction, comparing **(B-E)** all treated samples to the DMSO control or **(A+F)** all other samples to the 2-DG-treated sample (white bar). p-values are indicated as follows: < 0.05 = *, < 0.01 = **, < 0.001 = ***, < 0.0001 = ****.
(TIFF)

S15 Fig MPI expression and mannose-mediated restoration of viral protein expression. 24 h after seeding, A549 cells were infected with SC35M at an MOI of 5 or for 30 min and were incubated in the presence of the indicated concentrations of 2-DG and mannose or their solvents for a total of 8 h. Subsequently, protein lysates of triplicates were unified to yield sufficient protein amounts. Proteins were separated via SDS-PAGE. Visualization was done using primary antibodies against MPI (rabbit), PA (rabbit), M1 (mouse) and ERK2 (rabbit) and fluorescence-labelled anti-mouse (donkey) and anti-rabbit (donkey) secondary antibodies. **(A)** Depicted are representative protein bands from one out of three independent experiments. **(B-D)** Densitometric analyses were performed to quantify protein accumulation by first normalizing target proteins to the loading control ERK2 and then normalizing all other samples to **(B)** the mock-infected control or **(C+D)** to the infected but untreated sample. Depicted are the means *±* SD of three independent experiments. Statistical significances were determined via unpaired one-way ANOVA and Tukey’s correction, comparing all samples with each other. p-values are indicated as follows: < 0.05 = *, < 0.01 = **, < 0.001 = ***, < 0.0001 = ****.
(TIFF)

S16 Fig Gating strategy for the quantification of uninfected versus infected and living versus dead cells. A549 cells were infected with SC35M at an MOI of 0.01. Directly after the infection, cells were mock-treated or treated with 2-DG. 24 hpi cells were stained with a viability dye and an NP antibody and were quantified via flow cytometry. At first cells were pre-gated according to their FSC/SSC appearance. Then these cells were sub-classified to discriminate between uninfected and infected cells as well as living and dead cells. Representative dot plots are depicted to exemplify the gating strategy used for data analysis in S1A and S1B Fig.
(PNG)

S1 Table n-fold changes over control and statistical significances of Fig 6. (XLSX)

S2 Table n-fold changes over control and statistical significances of S13 Fig. (XLSX)
