# Supplementary figures and images for: Glycolytic interference blocks influenza A virus propagation by impairing viral polymerase-driven synthesis of genomic vRNA
Source: PLoS Pathog. 2023 Jul 13;19(7):e1010986. doi: 10.1371/journal.ppat.1010986 (PMC10343032; doi:10.1371/journal.ppat.1010986)

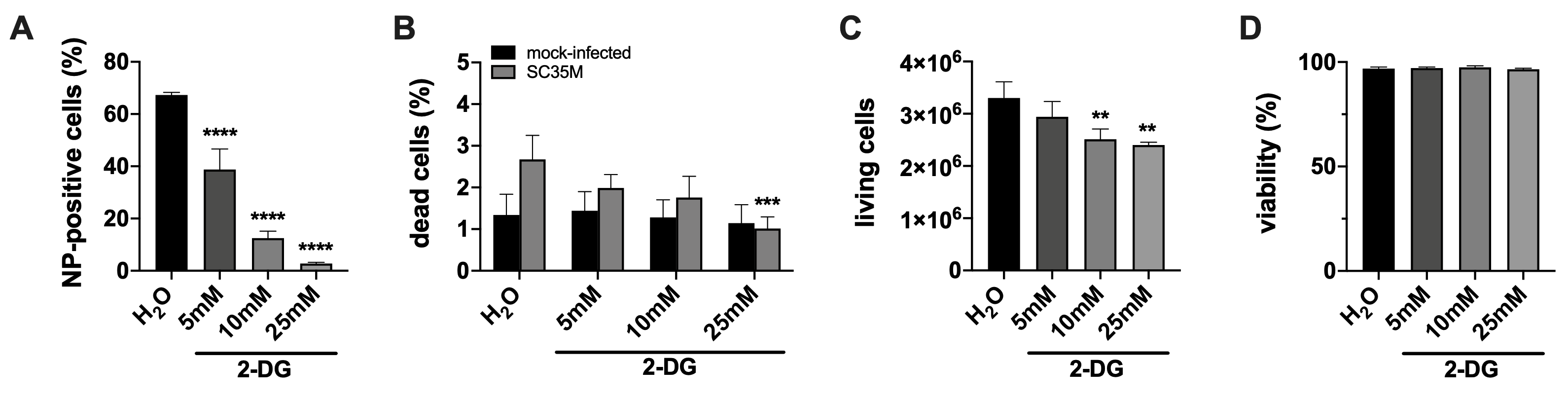

Supplement: S1 Fig — 24 h after seeding, A549 cells were infected with SC35M at an MOI of (A+B) 0.01 for 30 min or (C+D) remained uninfected and were incubated in the presence of the indicated concentrations of 2-DG or its solvent water for 24 h. Subsequently, cells were (A+B) stained with an NP antibody and a live/dead marker and were analyzed via flow cytometry or (C+D) were detached to assess the number of living cells as well as the viability via trypan blue exclusion in an automated cell counter. (A-D) Depicted are the means ± SD of three independent experiments with three biological replicates per condition and experiment. Statistical significances were determined via (A, C, D) unpaired one-way ANOVA and Dunnett’s correction, comparing all treated samples to the water control or (B) ordinary two-way ANOVA with Dunnett’s correction, comparing all treated samples of both groups to their respective water control. p-values are indicated as follows: < 0.05 = *, < 0.01 = **, < 0.001 = ***, < 0.0001 = ****. (TIFF) [file ppat.1010986.s001.tiff]

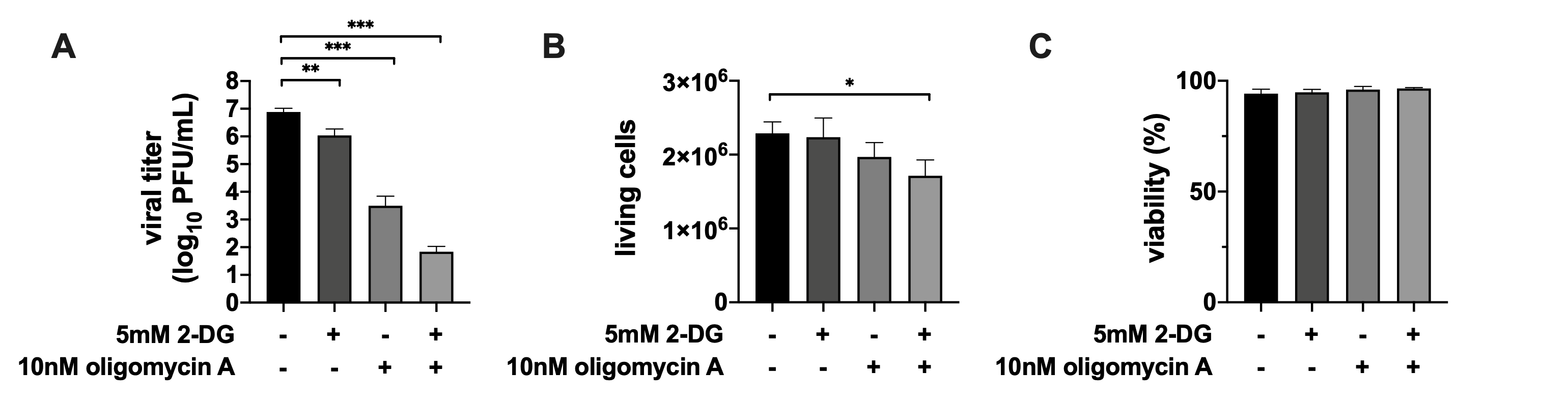

Supplement: S2 Fig — 24 h after seeding, A549 cells were infected with SC35M at an MOI of 0.001 for 30 min and were incubated in the presence of 25mM glucose and the indicated concentrations of 2-DG and/or oligomycin A or their solvents water and DMSO for 24 h. (A) Subsequently, supernatants were collected to determine viral titers via plaque assay and (B+C) cells were detached to assess the number of living cells as well as the viability via trypan blue exclusion in an automated cell counter. (A-C) Depicted are the means ± SD of three independent experiments with three biological replicates per condition and experiment. Statistical significances were determined via unpaired one-way ANOVA and Tukey’s correction, comparing all samples with each other. p-values are indicated as follows: < 0.05 = *, < 0.01 = **, < 0.001 = ***, < 0.0001 = ****. (TIFF) [file ppat.1010986.s002.tiff]

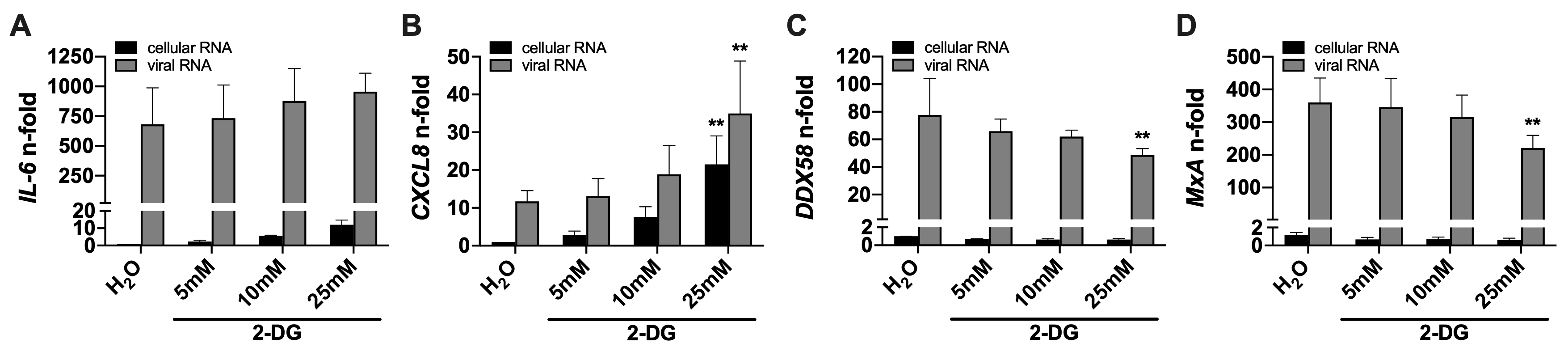

Supplement: S3 Fig — 24 h after seeding, uninfected cells were transfected with cellular or viral RNA and treated with the indicated 2-DG concentrations for 6 h. Subsequently, cells were lysed, their RNA isolated and cDNA synthesized using oligo(dT) primers to transcribe mRNA. Real-time qPCR was performed with two technical replicates per sample and values of all other samples were normalized to the unstimulated water control. Additionally, all results were normalized to a GAPDH control. Depicted are the means ± SD of three independent experiments with three biological replicates per condition and experiment. Statistical significances were determined via ordinary two-way ANOVA with Dunnett’s correction, comparing all treated samples of both groups to their respective water control. p-values are indicated as follows: < 0.05 = *, < 0.01 = **, < 0.001 = ***, < 0.0001 = ****. (TIFF) [file ppat.1010986.s003.tiff]

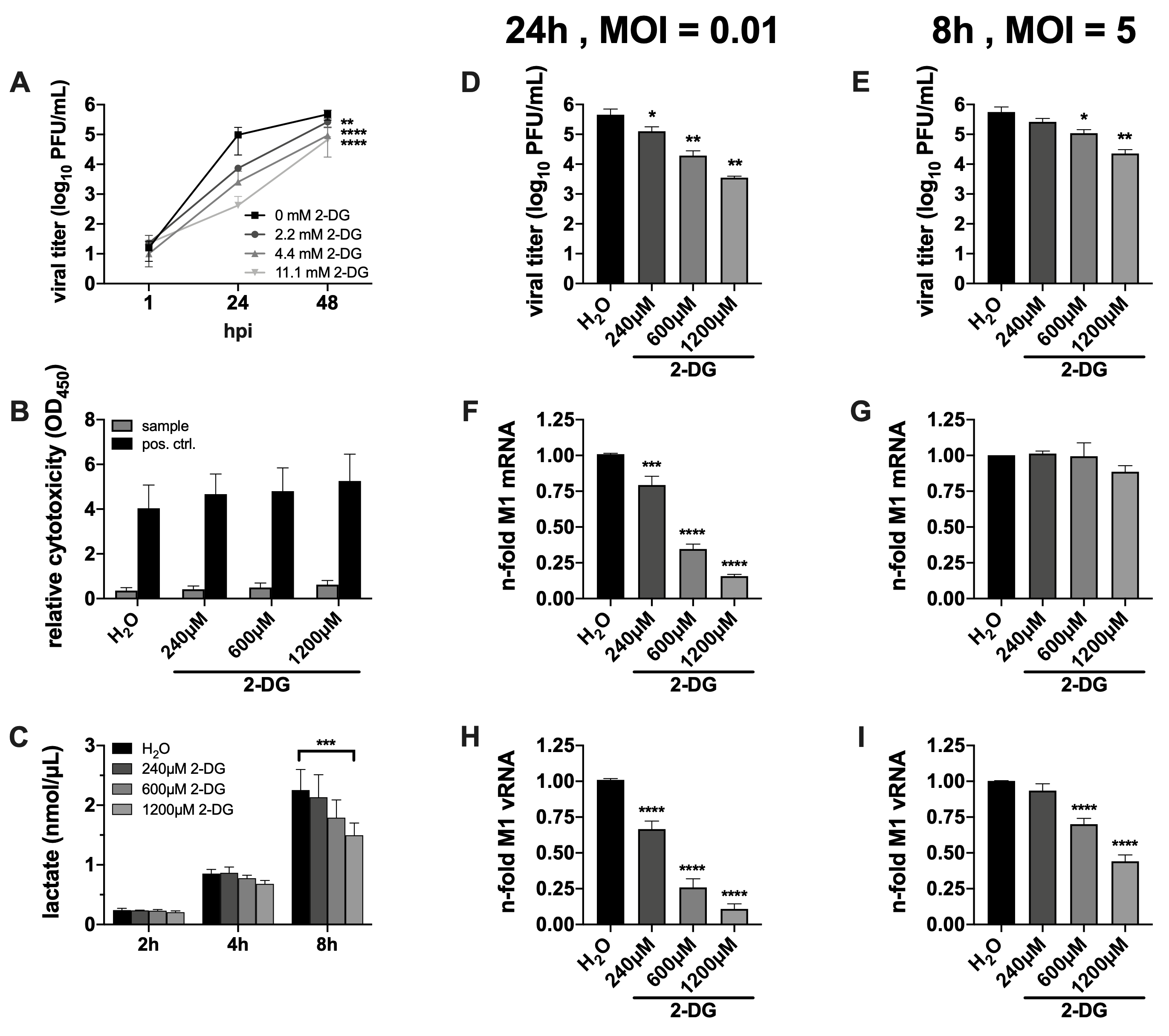

Supplement: S4 Fig — (A) Human lung explants were infected with 2 x 10^5^ SC35M particles for 30 min. Afterwards they were incubated with 11.1 mM glucose and the indicated concentrations of 2-DG and supernatants were collected 1, 24 and 48 hpi to determine viral titers via plaque assay. (B-I) After reaching ≈ 90% confluency (B) uninfected HBEpCs were treated with the indicated concentrations of 2-DG or its solvent water for 24 h. Afterwards the supernatants were used to perform LDH assays to determine the relative cytotoxicity of the treatment. (C-I) HBEpCs were infected with SC35M at an MOI of (C) 1, (D, F, H) 0.01 or (E, G, I) 5 for 30 min and were incubated with 6 mM glucose and the indicated concentrations of 2-DG for a total of (D, F, H) 24 h or (E, G, I) 8 h. Subsequently, (C-E) supernatants were used to (C) perform lactate assays in order to indirectly assess the glycolytic activity and (D+E) determine viral titers via plaque assay. (F-I) Additionally, cells were lysed, their RNA isolated and cDNA synthesized using either (F+H) oligo(dT) primers to transcribe mRNA or (H+I) fluA uni12 primers to transcribe vRNA. Real-time qPCR was performed with two technical replicates per sample and values of treated samples were normalized to the water control. In case of mRNA detection, all results were additionally normalized to a GAPDH control. Depicted are the means ± SD of three independent experiments with three biological replicates per condition and experiment. Statistical significances were determined via (A-C) ordinary two-way ANOVA and Dunnett’s correction, comparing each treated sample to its respective water control. (D-I) Other significances were determined via unpaired one-way ANOVA and Dunnett’s correction, comparing all treated samples to the water control. p-values are indicated as follows: < 0.05 = *, < 0.01 = **, < 0.001 = ***, < 0.0001 = ****. (TIFF) [file ppat.1010986.s004.tiff]

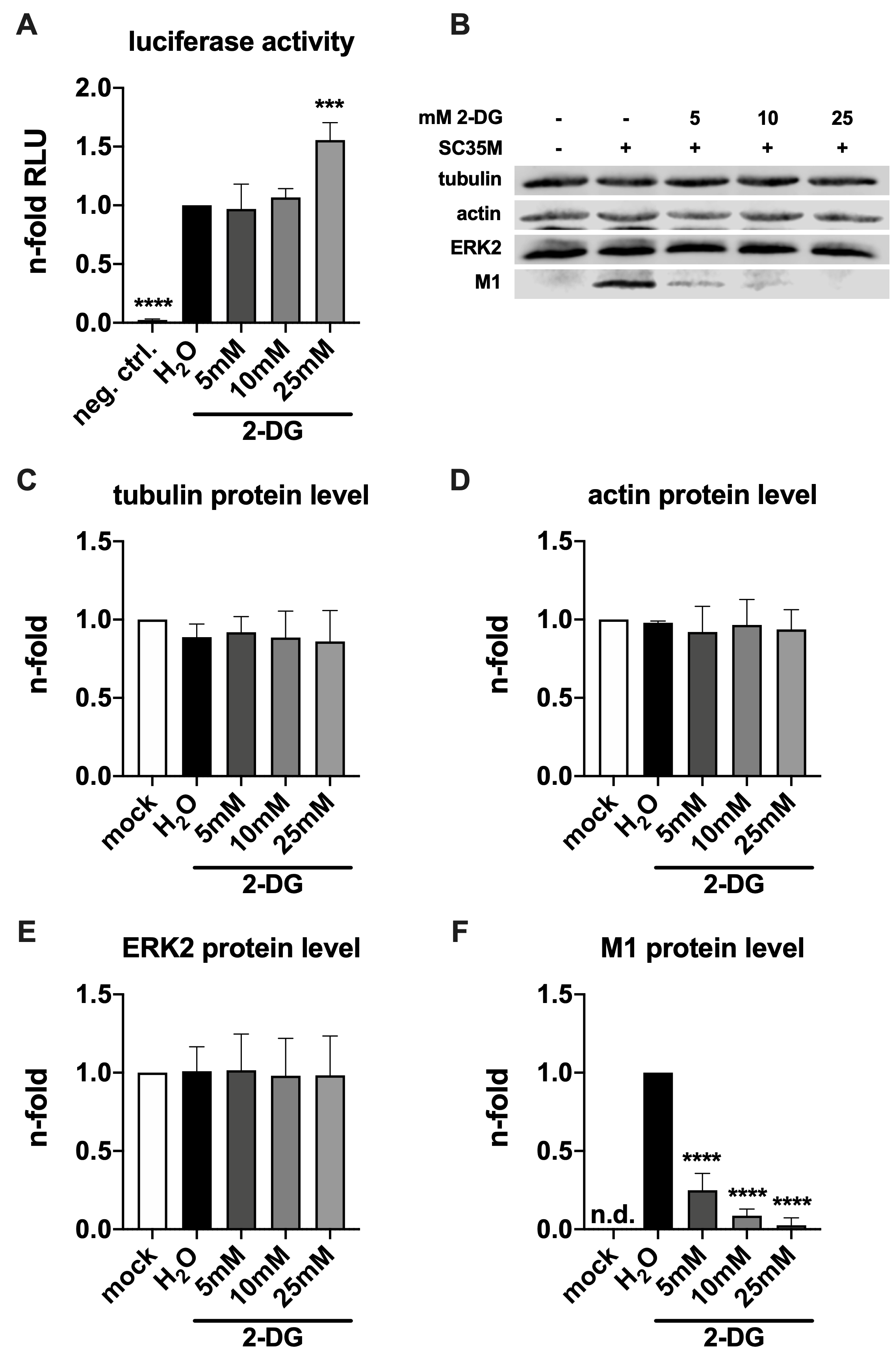

Supplement: S5 Fig — (A) 24 h after seeding, HEK293T cells were transfected with an empty vector or a plasmid containing the Renilla luciferase gene which is under the control of a constitutive herpes simplex virus thymidine kinase promoter. Subsequently, the cells were incubated with the shown 2-DG concentrations. After 24 h, cells were lysed and the n-fold of relative light units (RLU) in comparison to the water control was measured via luciferase assay. Depicted are the means ± SD of three independent experiments with three biological replicates per condition and experiment. Statistical significances were determined via unpaired one-way ANOVA and Dunnett’s correction, comparing all treated samples to the water control. (B) 24 h after seeding, A549 cells were infected with SC35M at an MOI of 0.001 for 30 min and were incubated with 25 mM glucose and the indicated concentrations of 2-DG for a total of 24 h. Protein lysates of triplicates were unified to yield sufficient protein amounts. Proteins were separated via SDS-PAGE. Visualization was done using primary antibodies against α-tubulin (mouse), β-actin (mouse), ERK2 (rabbit) and M1 (mouse) and fluorescence-labelled anti-mouse (donkey) and anti-rabbit (donkey) secondary antibodies. Depicted are representative protein bands from one out of three independent experiments. (C-F) Densitometric analyses were performed to quantify protein accumulation. The n-folds were calculated in regard to (C-D) the mock control or (F) the infected and untreated sample. Depicted are the means ± SD of three independent experiments. Statistical significances were determined via unpaired one-way ANOVA and Dunnett’s correction, comparing all other samples to (C-D) the mock control or (F) the infected and untreated sample. p-values are indicated as follows: < 0.05 = *, < 0.01 = **, < 0.001 = ***, < 0.0001 = ****. (TIFF) [file ppat.1010986.s005.tiff]

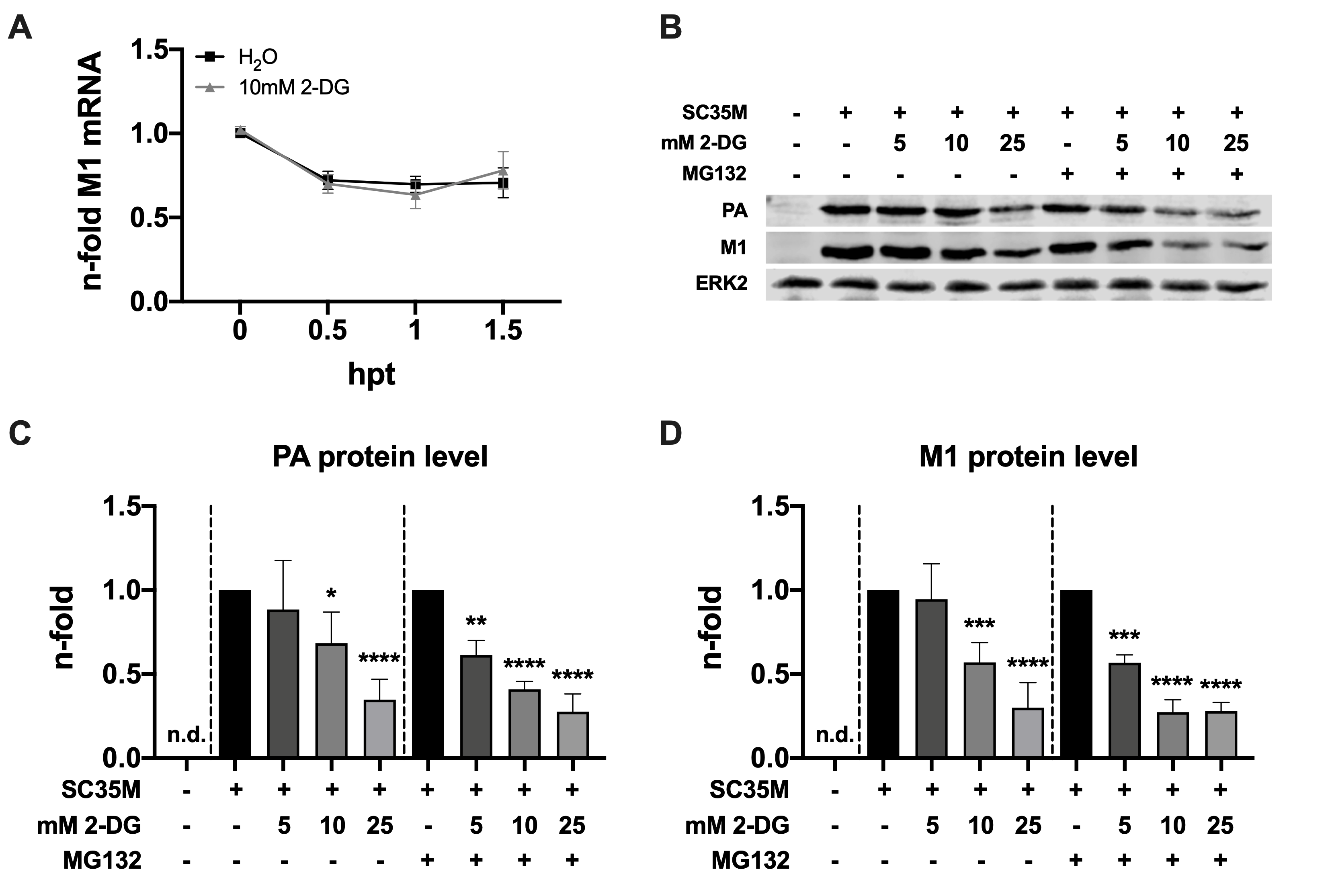

Supplement: S6 Fig — 24 h after seeding, A549 cells were infected with SC35M at an MOI of 5 for 30 min and were incubated with 25 mM glucose and the indicated concentrations of 2-DG. (A) 6 hpi media were replaced with the same media containing actinomycin D (10 μg/mL) and cells were incubated in it for the depicted time points. Subsequently, cells were lysed, their RNA isolated and cDNA synthesized using oligo(dT) primers. Real-time qPCR was performed with two technical replicates per sample and values of treated samples were normalized to the water control. All results were additionally normalized to a GAPDH control. Depicted are the means ± SD of three independent experiments with three biological replicates per condition and experiment. Statistical significances were determined via ordinary two-way ANOVA and Sidak’s correction, comparing the samples of a common time point with each other. (B) 2 hpi MG132 was added to the media (20 μM) and cells were incubated with it for another 6 h. Protein lysates of triplicates were unified to yield sufficient protein amounts. Proteins were separated via SDS-PAGE. Visualization was done using primary antibodies against PA (rabbit), M1 (mouse) and ERK2 (rabbit) and fluorescence-labelled anti-mouse (donkey) and anti-rabbit (donkey) secondary antibodies. Depicted are representative protein bands from one out of three independent experiments. (C+D) Densitometric analyses were performed to quantify protein accumulation by first normalizing PA and M1 to the loading control ERK2 and then normalizing all other samples to the infected but untreated sample. MG132(+) and MG132(-) samples were normalized independently. Depicted are the means ± SD of three independent experiments. Statistical significances were determined via unpaired one-way ANOVA and Dunnett’s correction, comparing all other samples to the infected but untreated sample (second/sixth lane). p-values are indicated as follows: < 0.05 = *, < 0.01 = **, < 0.001 = ***, < 0.0001 = ****. (TIFF) [file ppat.1010986.s006.tiff]

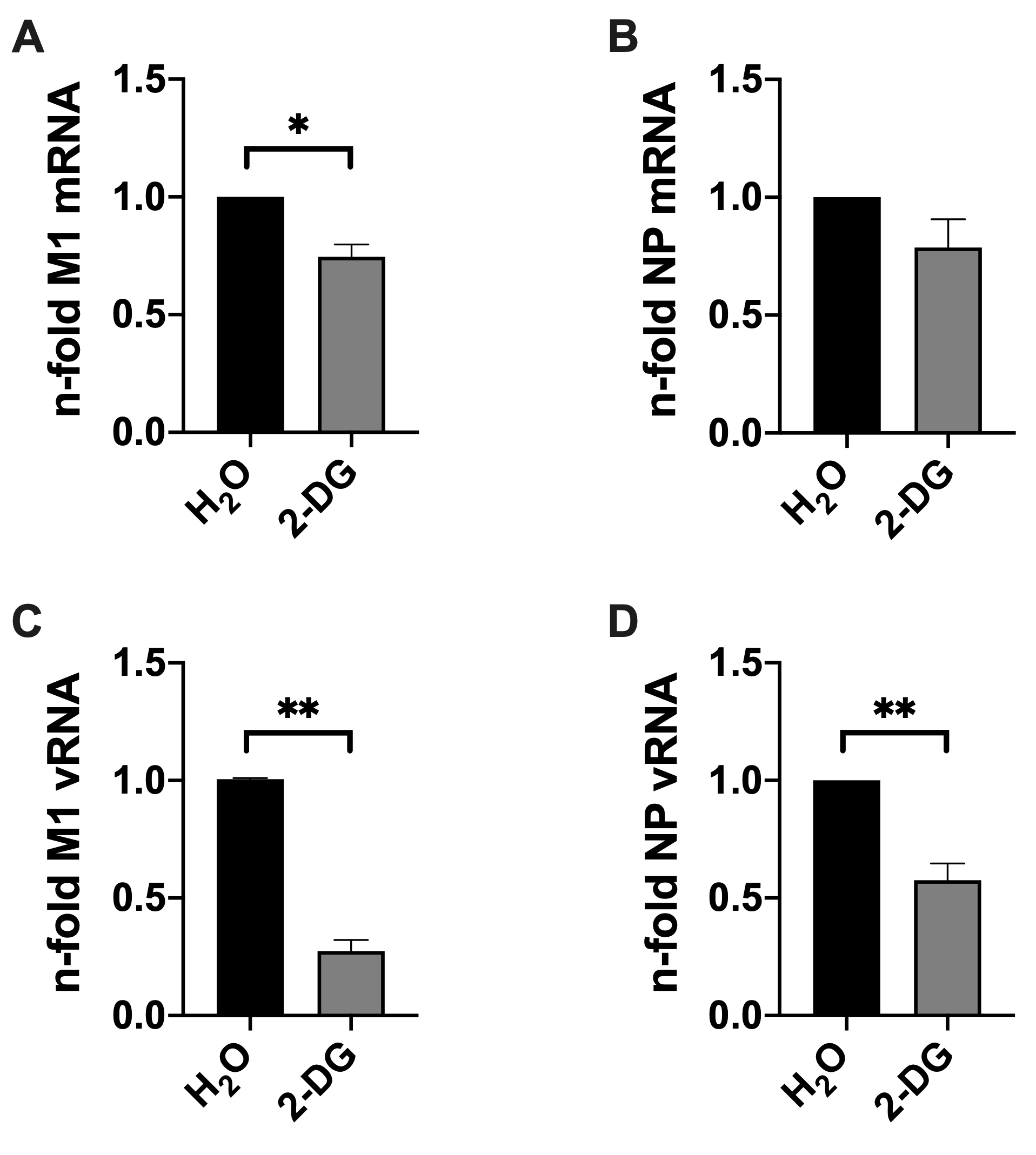

Supplement: S7 Fig — 24 h after seeding, Calu-3 cells were infected with SC35M at an MOI of 5 for 30 min and were incubated with 25 mM glucose and 10 mM 2-DG or its solvent water for a total of 8 h. Subsequently, cells were lysed, their RNA isolated and cDNA synthesized using either (A) oligo(dT) primers, (C) fluA uni12 primers or (B+D) specific primers to transcribe mRNA or vRNA of M1 and NP. Real-time qPCR was performed with two technical replicates per sample and values of treated samples were normalized to the water control. In case of mRNA detection, all results were additionally normalized to a GAPDH control. Depicted are the means ± SD of three independent experiments with three biological replicates per condition and experiment. Statistical significances were determined via unpaired t-test with Welch’s correction. p-values are indicated as follows: < 0.05 = *, < 0.01 = **, < 0.001 = ***, < 0.0001 = ****. (TIFF) [file ppat.1010986.s007.tiff]

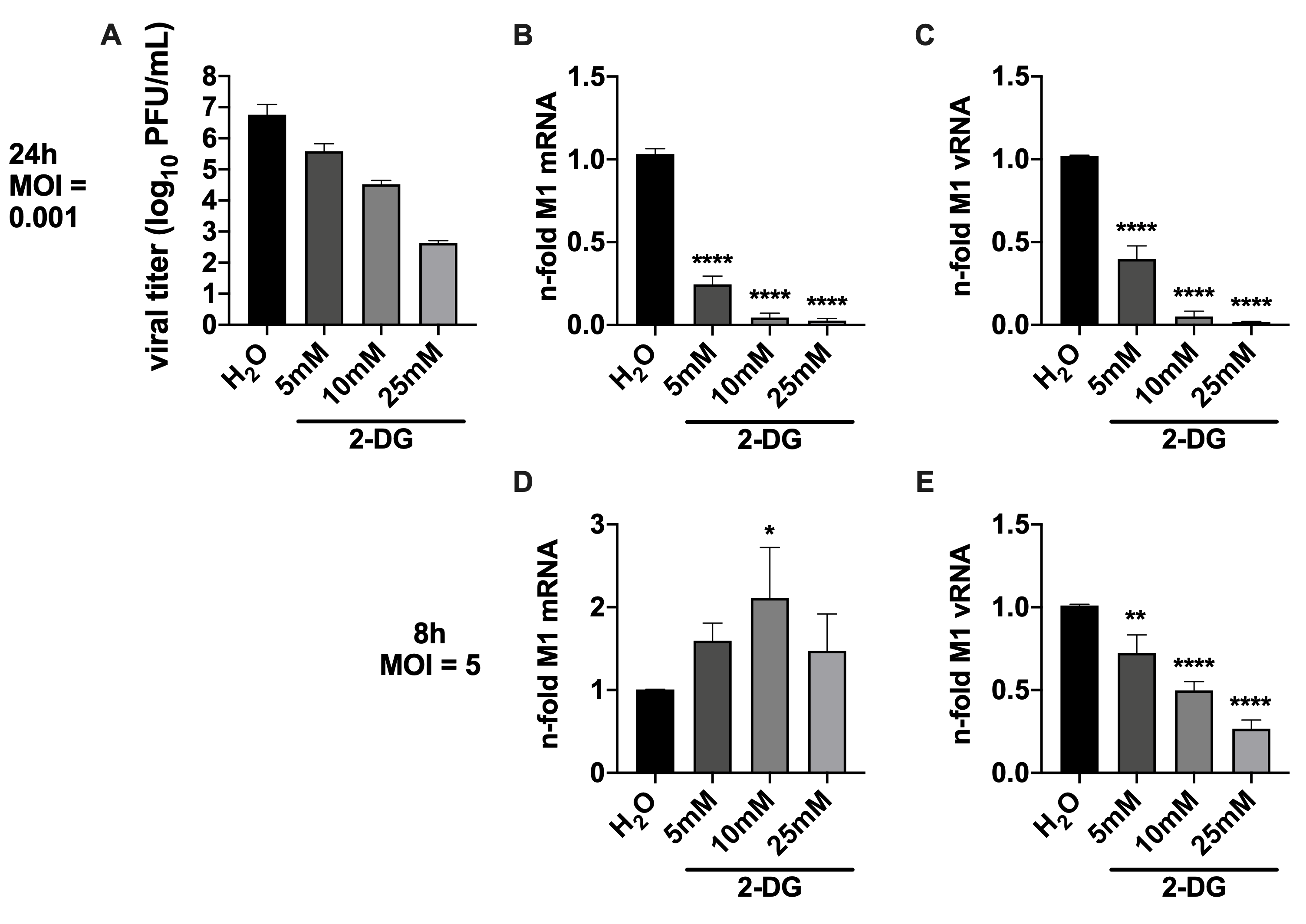

Supplement: S8 Fig — 24 h after seeding, A549 cells were infected with Pan/99 at the depicted MOIs for 30 min and were incubated with the indicated concentrations of 2-DG or its solvent water for a total of (A-C) 24 h or (D+E) 8 h. Subsequently, (A) supernatants were collected to determine viral titers via plaque assay or (B-E) cells were lysed, their RNA isolated and cDNA synthesized using either (B+D) oligo(dT) primers to transcribe mRNA or (C+E) fluA uni12 primers to transcribe vRNA. Real-time qPCR was performed with two technical replicates per sample and values of treated samples were normalized to the water control. In case of mRNA detection, all results were additionally normalized to a GAPDH control. Depicted are the means ± SD of three independent experiments with three biological replicates per condition and experiment. Statistical significances were determined via unpaired one-way ANOVA and Dunnett’s correction, comparing all treated samples to the water control. p-values are indicated as follows: < 0.05 = *, < 0.01 = **, < 0.001 = ***, < 0.0001 = ****. (TIFF) [file ppat.1010986.s008.tiff]

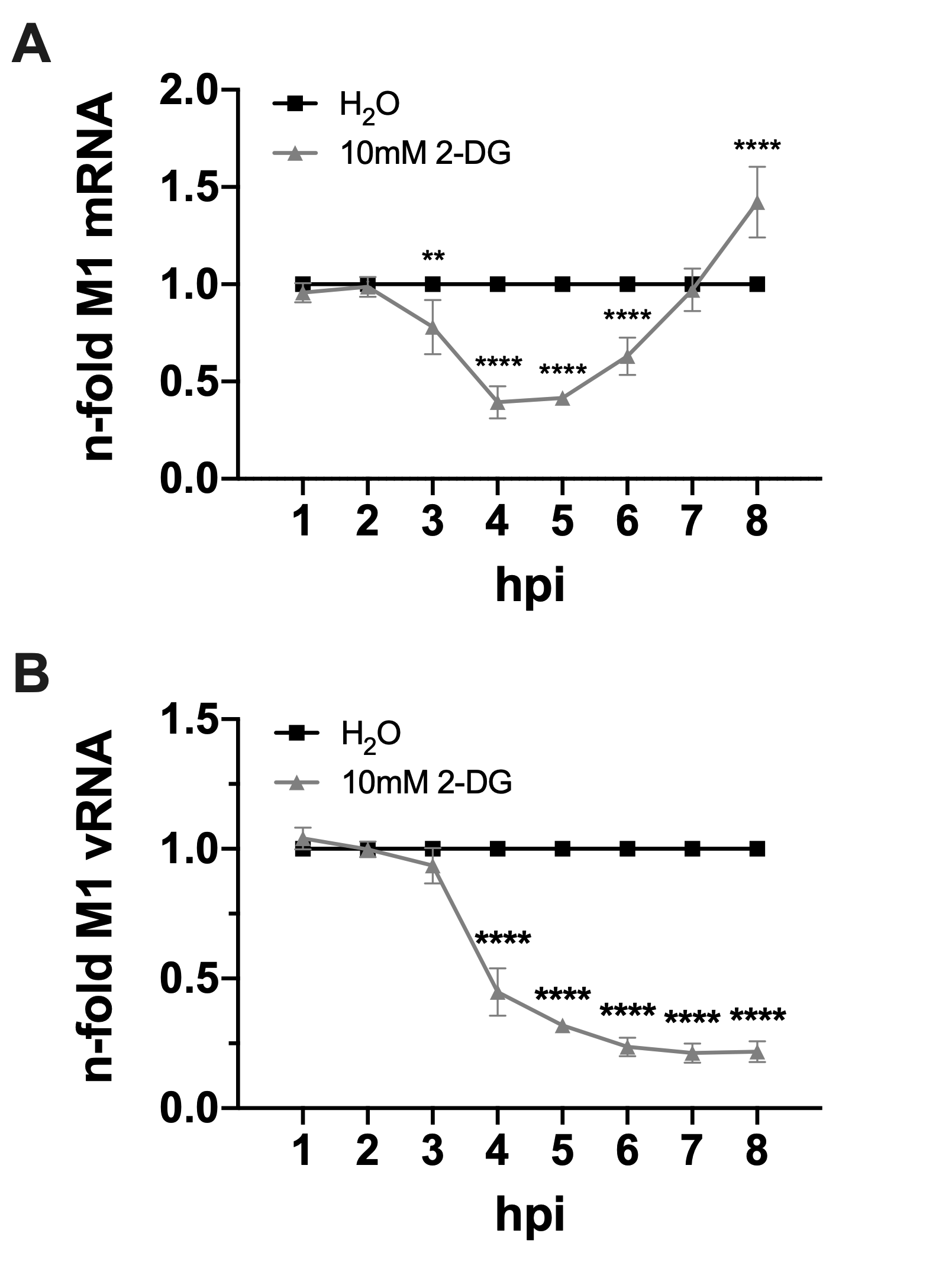

Supplement: S9 Fig — 24 h after seeding, A549 cells were infected with SC35M at an MOI of 5 for 30 min and were incubated without or with 10 mM 2-DG in the presence of 25 mM glucose for a total of 8 h. (A+B) Each hour cells were lysed, their RNA isolated and cDNA synthesized using (A) oligo(dT) primers or (B) fluA uni12 primers. Real-time qPCR was performed with two technical replicates per sample. The raw data are the same as in Fig 4A and 4B but the values of each time point were normalized to the water control of the same time point. Depicted are the means ± SD of three independent experiments with three biological replicates per condition and experiment. Statistical significances were determined via ordinary two-way ANOVA and Sidak’s correction, comparing the treated sample of each time point to its respective water control. p-values are indicated as follows: < 0.05 = *, < 0.01 = **, < 0.001 = ***, < 0.0001 = ****. (TIFF) [file ppat.1010986.s009.tiff]

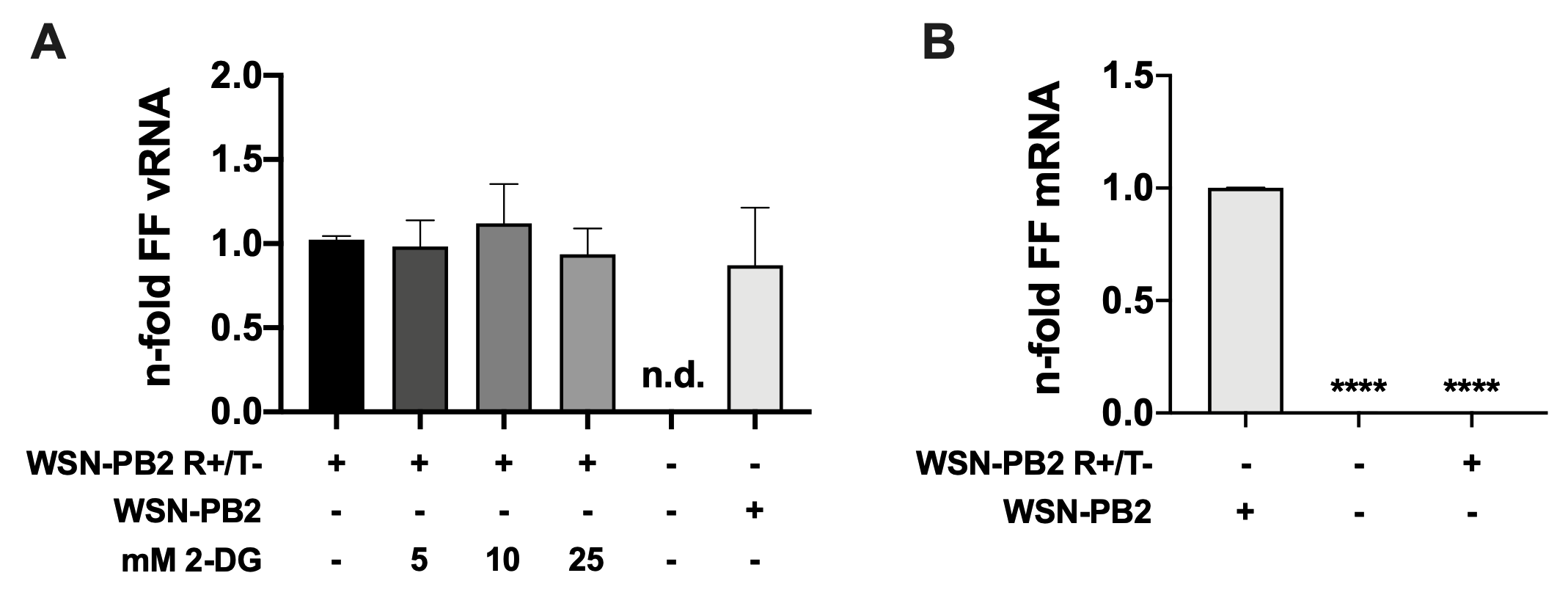

Supplement: S10 Fig — 24 h after seeding, HEK293T cells were transfected with plasmids encoding NP, PA, PB1 and PB2 R+/T- of WSN as well as either a (A) cRNA-like or (B) vRNA-like template of the Firefly (FF) luciferase. The negative control was transfected with an empty vector instead of PB2 while the positive control was transfected with wild type PB2 instead of PB2 R+/T-. 4 h later the transfection solution was replaced with medium containing 25 mM glucose and the indicated concentrations of 2-DG for another 20 h. Subsequently, cells were lysed, their RNA isolated and cDNA synthesized using specific primers to transcribe (A) vRNA and (B) mRNA of FF. Real-time qPCR was performed with two technical replicates per sample and values of treated samples were normalized to the water control. Depicted are the means ± SD of three independent experiments with three biological replicates per condition and experiment. Statistical significances were determined via unpaired one-way ANOVA and Dunnett’s correction, comparing all other samples to the (A) water control or (B) the PB2 positive control. p-values are indicated as follows: < 0.05 = *, < 0.01 = **, < 0.001 = ***, < 0.0001 = ****. (TIFF) [file ppat.1010986.s010.tiff]

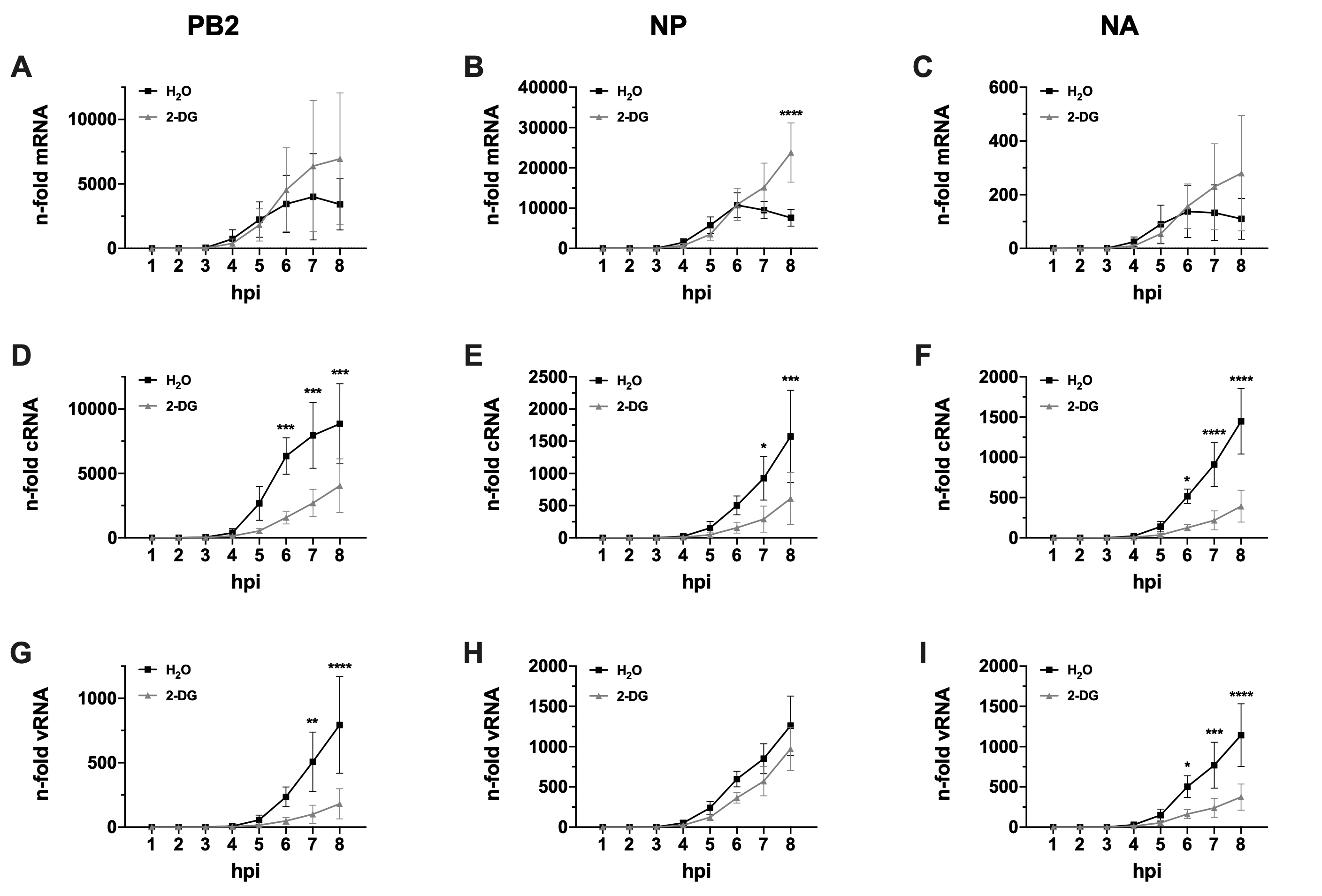

Supplement: S11 Fig — 24 h after seeding, A549 cells were infected with SC35M at an MOI of 5 for 30 min and were afterwards incubated without or with 10 mM 2-DG in the presence of 25 mM glucose for a maximum of 8 h. Each hour cells were lysed, their RNA isolated and cDNA synthesized using specific primers to transcribe mRNA, cRNA and vRNA of the SC35M gene segments 1 (PB2), 5 (NP) and 6 (NA). Real-time qPCR was performed with two technical replicates per sample. All values were normalized to the water control 1 hpi. Depicted are the means ± SD of three independent experiments with three biological replicates per condition and experiment. Statistical significances were determined via ordinary two-way ANOVA and Sidak’s correction, comparing the treated sample of each time point to its respective water control. p-values are indicated as follows: < 0.05 = *, < 0.01 = **, < 0.001 = ***, < 0.0001 = ****. (TIFF) [file ppat.1010986.s011.tiff]

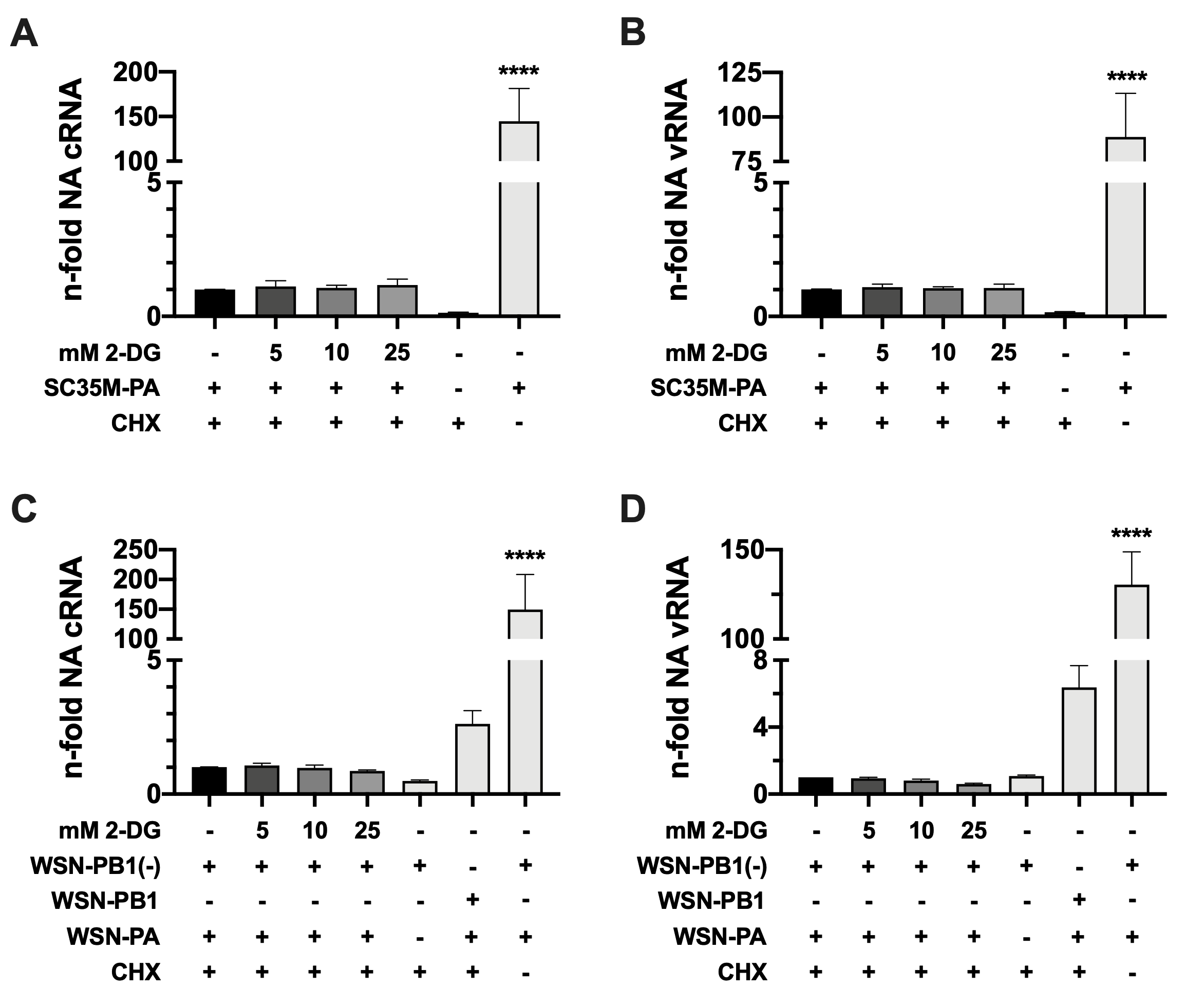

Supplement: S12 Fig — 24 h after seeding, HEK293T cells were transfected with plasmids containing (A+B) the SC35M sequences of PA, PB1, PB2 and NP or (C+D) the WSN sequences of PA, PB1 or PB1(-), PB2 and NP. 4 h later the transfection solution was replaced with fresh medium for another 20 h. Subsequently, cells were infected with SC35M at an MOI of 5 for 30 min and were incubated with the indicated concentrations of 2-DG and 100 μg/mL cycloheximide. A negative control was previously transfected with an empty vector instead of PA while a positive control was not treated with cycloheximide. 6 hpi, cells were lysed, their RNA isolated and cDNA synthesized using specific primers to transcribe cRNA and vRNA of the (A+B) SC35M or (C+D) WSN gene segment 6 (NA). Real-time qPCR was performed with two technical replicates per sample. Statistical significances were determined via unpaired one-way ANOVA and Dunnett’s correction, comparing all other samples to the respective water control. p-values are indicated as follows: < 0.05 = *, < 0.01 = **, < 0.001 = ***, < 0.0001 = ****. (TIFF) [file ppat.1010986.s012.tiff]

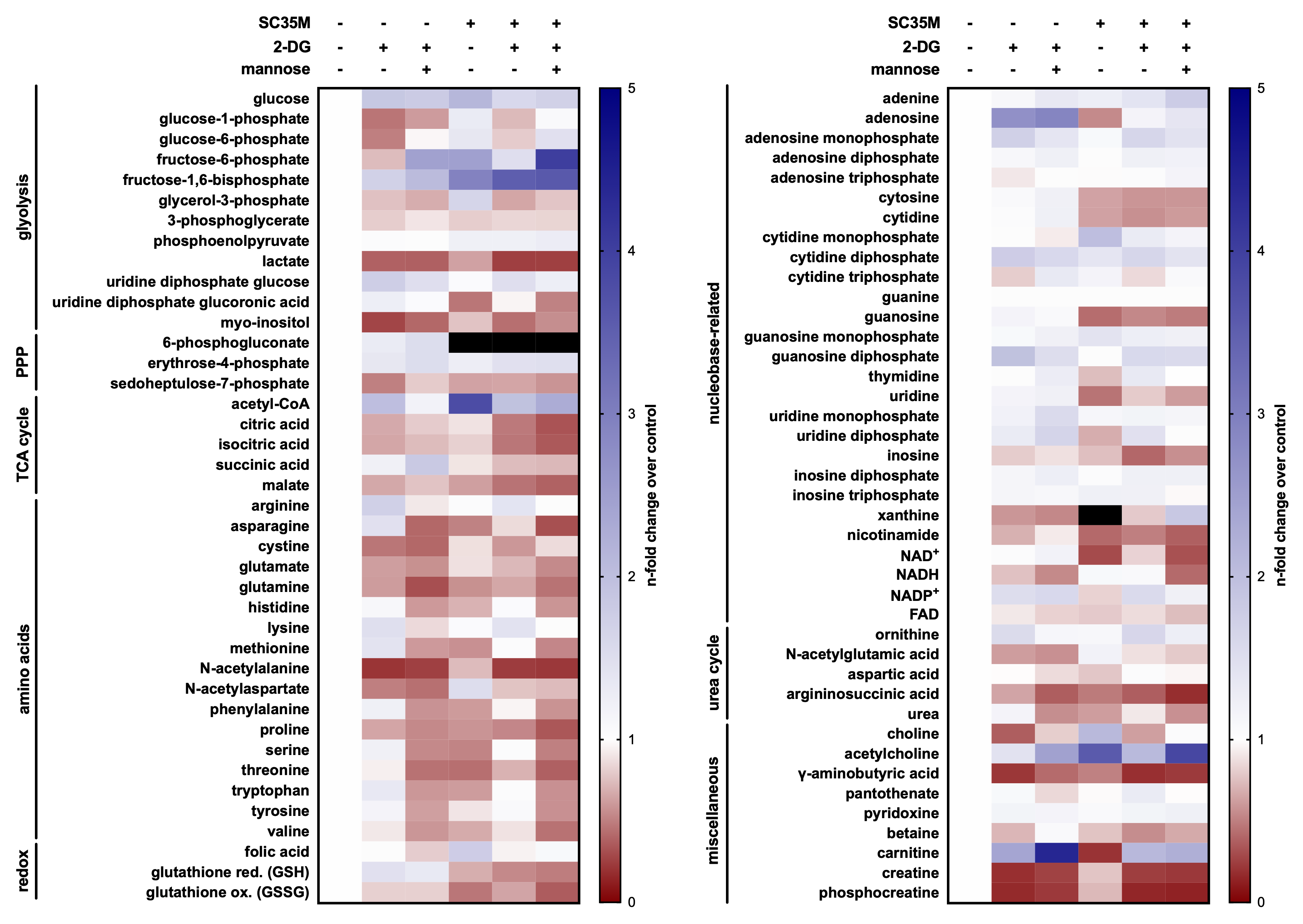

Supplement: S13 Fig — A549 cells were mock-infected or infected with SC35M at an MOI of 0.1 and were subsequently incubated in DMEM (containing 25 mM glucose) with or without 10 mM 2-DG and 1 mM mannose as indicated. 24 hpi metabolic activity was quenched and intracellular metabolites were relatively quantified via HILIC-MS/MS. All values have been normalized to the uninfected and untreated control (left column). Darker shades of blue indicate a higher and darker shades of red indicate a lower n-fold of the respective metabolite compared to the control. Black indicates increases higher than 5-fold compared to the control. Depicted are the means of three independent experiments with three biological replicates per condition and experiment. Statistical significances were determined via ordinary two-way ANOVA and Dunnett’s correction, comparing all samples to their respective uninfected and untreated control. The n-folds and p-values are presented in S2 Table. (TIFF) [file ppat.1010986.s013.tiff]

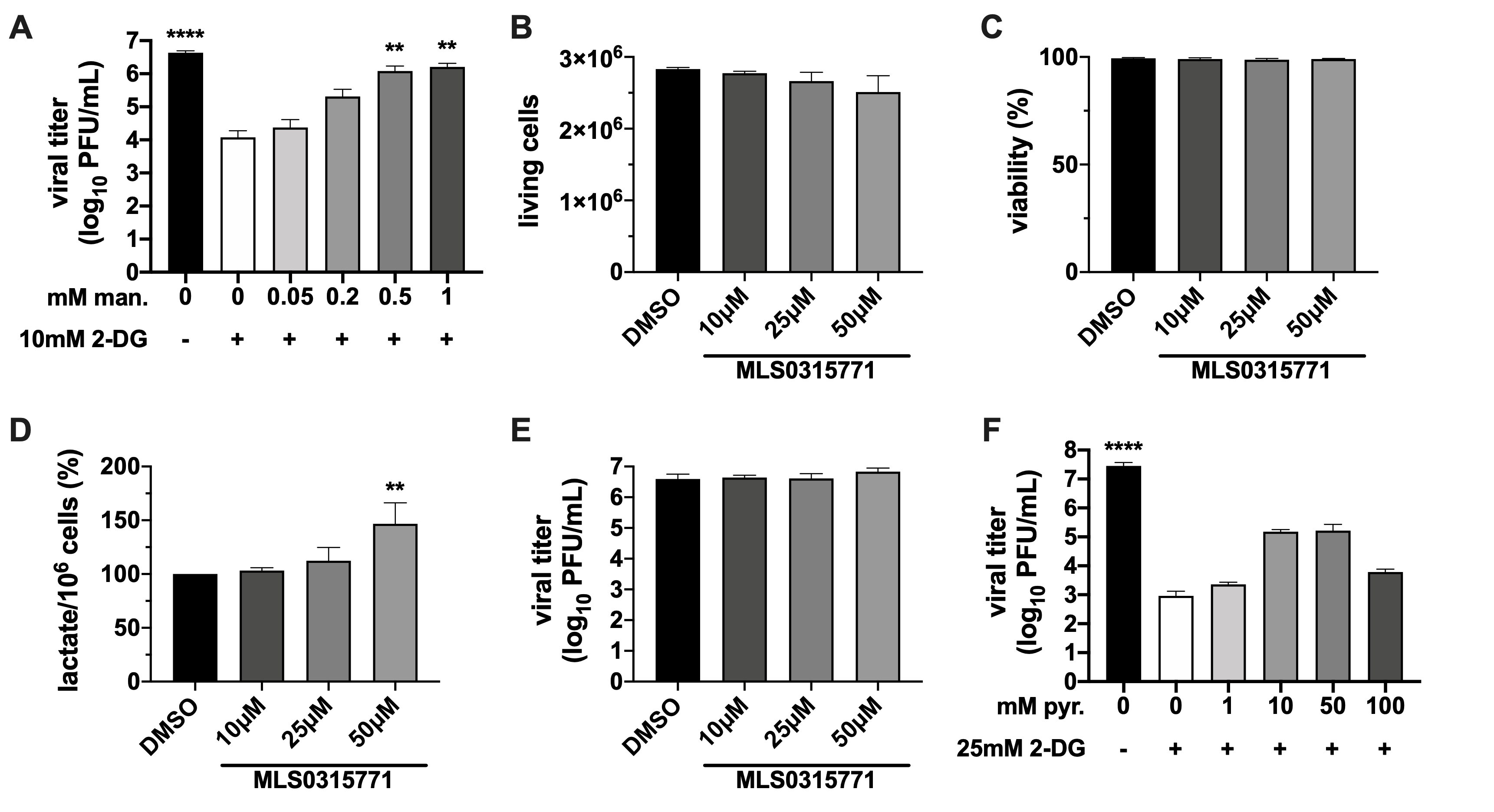

Supplement: S14 Fig — 24 h after seeding, A549 cells were infected with SC35M at an MOI of 0.001 or for 30 min and were incubated in the presence of the indicated concentrations of metabolites and inhibitors or their solvents for a total of 24 h. Subsequently, (A, D-F) supernatants were collected to determine (A, E, F) viral titers via plaque assay and (D) extracellular lactate concentrations via lactate assay or (B+C) cells were detached to assess the number of living cells and the viability via trypan blue exclusion and an automated cell counter. Depicted are the means ± SD of three independent experiments with three biological replicates per condition and experiment. Statistical significances were determined via unpaired one-way ANOVA and Dunnett’s correction, comparing (B-E) all treated samples to the DMSO control or (A+F) all other samples to the 2-DG-treated sample (white bar). p-values are indicated as follows: < 0.05 = *, < 0.01 = **, < 0.001 = ***, < 0.0001 = ****. (TIFF) [file ppat.1010986.s014.tiff]

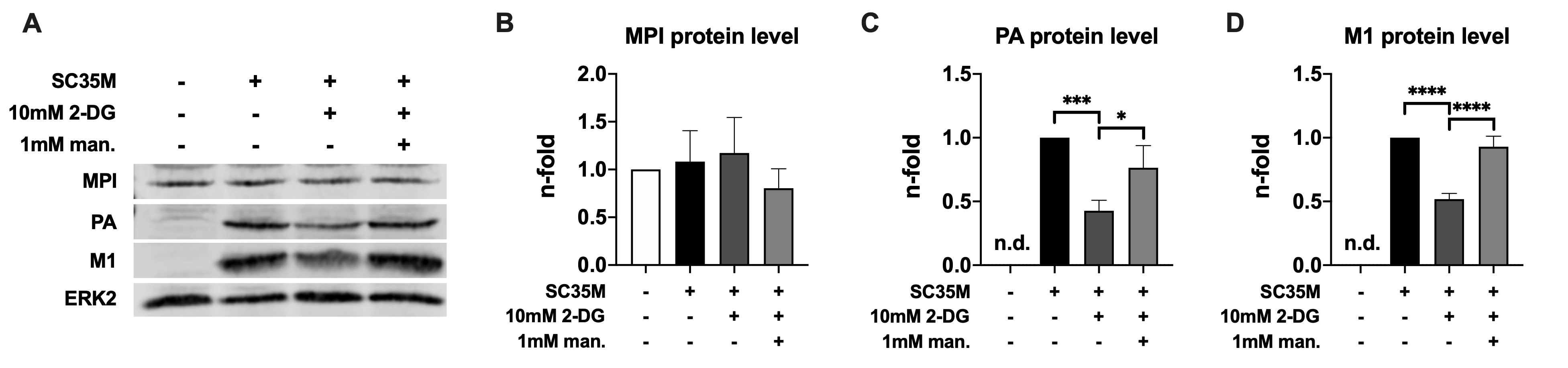

Supplement: S15 Fig — 24 h after seeding, A549 cells were infected with SC35M at an MOI of 5 or for 30 min and were incubated in the presence of the indicated concentrations of 2-DG and mannose or their solvents for a total of 8 h. Subsequently, protein lysates of triplicates were unified to yield sufficient protein amounts. Proteins were separated via SDS-PAGE. Visualization was done using primary antibodies against MPI (rabbit), PA (rabbit), M1 (mouse) and ERK2 (rabbit) and fluorescence-labelled anti-mouse (donkey) and anti-rabbit (donkey) secondary antibodies. (A) Depicted are representative protein bands from one out of three independent experiments. (B-D) Densitometric analyses were performed to quantify protein accumulation by first normalizing target proteins to the loading control ERK2 and then normalizing all other samples to (B) the mock-infected control or (C+D) to the infected but untreated sample. Depicted are the means ± SD of three independent experiments. Statistical significances were determined via unpaired one-way ANOVA and Tukey’s correction, comparing all samples with each other. p-values are indicated as follows: < 0.05 = *, < 0.01 = **, < 0.001 = ***, < 0.0001 = ****. (TIFF) [file ppat.1010986.s015.tiff]

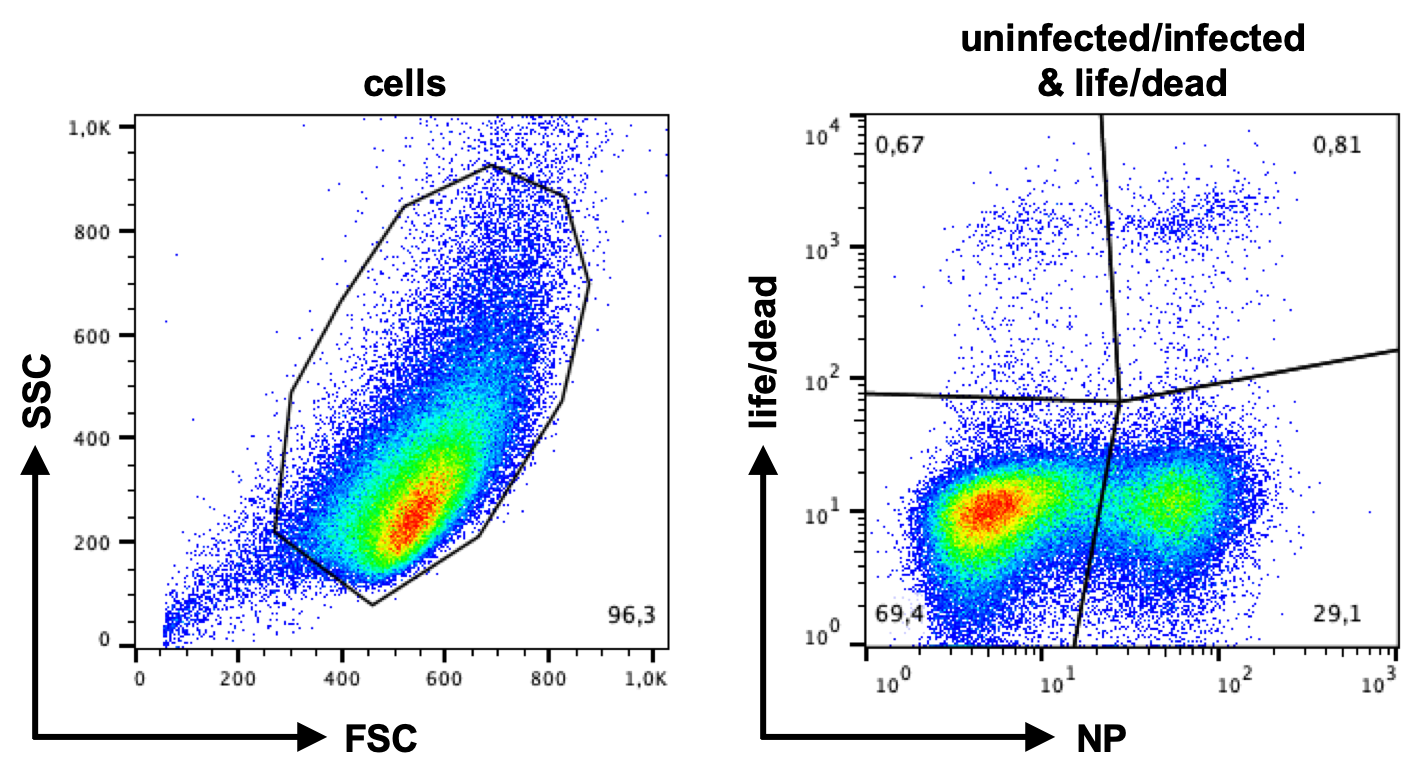

Supplement: S16 Fig — A549 cells were infected with SC35M at an MOI of 0.01. Directly after the infection, cells were mock-treated or treated with 2-DG. 24 hpi cells were stained with a viability dye and an NP antibody and were quantified via flow cytometry. At first cells were pre-gated according to their FSC/SSC appearance. Then these cells were sub-classified to discriminate between uninfected and infected cells as well as living and dead cells. Representative dot plots are depicted to exemplify the gating strategy used for data analysis in S1A and S1B Fig. (PNG) [file ppat.1010986.s016.png]
